# Altered host protease determinants for SARS-CoV-2 Omicron

**DOI:** 10.1126/sciadv.add3867

**Published:** 2023-01-20

**Authors:** Jasper Fuk-Woo Chan, Xiner Huang, Bingjie Hu, Yue Chai, Hongyu Shi, Tianrenzheng Zhu, Terrence Tsz-Tai Yuen, Yuanchen Liu, Huan Liu, Jialu Shi, Lei Wen, Huiping Shuai, Yuxin Hou, Chaemin Yoon, Jian-Piao Cai, Anna Jinxia Zhang, Jie Zhou, Feifei Yin, Shuofeng Yuan, Bao-Zhong Zhang, Melinda A. Brindley, Zheng-Li Shi, Kwok-Yung Yuen, Hin Chu

**Affiliations:** ^1^ State Key Laboratory of Emerging Infectious Diseases, Department of Microbiology and Carol Yu Centre for Infection, School of Clinical Medicine, Li Ka Shing Faculty of Medicine, The University of Hong Kong, Pokfulam, Hong Kong Special Administrative Region, People’s Republic of China.; ^2^ Department of Infectious Disease and Microbiology, The University of Hong Kong-Shenzhen Hospital, Shenzhen, Guangdong Province, People’s Republic of China.; ^3^ Centre for Virology, Vaccinology, and Therapeutics, Hong Kong Science and Technology Park, Hong Kong Special Administrative Region, People’s Republic of China.; ^4^ Department of Microbiology, Queen Mary Hospital, Pokfulam, Hong Kong, Special Administrative Region, People’s Republic of China.; ^5^ Academician Workstation of Hainan Province, Hainan Medical University–The University of Hong Kong Joint Laboratory of Tropical Infectious Diseases, Hainan Medical University, Haikou, Hainan Province, People’s Republic of China; and The University of Hong Kong, Pokfulam, Hong Kong Special Administrative Region, People’s Republic of China.; ^6^ Guangzhou Laboratory, Guangdong Province, China.; ^7^ Louis V. Gerstner Jr. Graduate School of Biomedical Sciences, Memorial Sloan Kettering Cancer Center, NY, New York, USA.; ^8^ Key Laboratory of Tropical Translational Medicine of Ministry of Education, Hainan Medical University, Haikou, Hainan Province, China.; ^9^ CAS Key Laboratory of Quantitative Engineering Biology, Shenzhen Institute of Synthetic Biology, Shenzhen Institutes of Advanced Technology, Chinese Academy of Sciences, Shenzhen 518055, People’s Republic of China.; ^10^ Department of Infectious Diseases and Department of Population Health, College of Veterinary Medicine, University of Georgia, Athens, GA 30602, USA.; ^11^ CAS Key Laboratory of Special Pathogens and Biosafety, Chinese Academy of Sciences, Wuhan Institute of Virology, Wuhan, Hubei, People’s Republic of China.

## Abstract

Successful severe acute respiratory syndrome coronavirus 2 (SARS-CoV-2) infection requires proteolytic cleavage of the viral spike protein. While the role of the host transmembrane protease serine 2 in SARS-CoV-2 infection is widely recognized, the involvement of other proteases capable of facilitating SARS-CoV-2 entry remains incompletely explored. Here, we show that multiple members from the membrane-type matrix metalloproteinase (MT-MMP) and a disintegrin and metalloproteinase families can mediate SARS-CoV-2 entry. Inhibition of MT-MMPs significantly reduces SARS-CoV-2 replication in vitro and in vivo. Mechanistically, we show that MT-MMPs can cleave SARS-CoV-2 spike and angiotensin-converting enzyme 2 and facilitate spike-mediated fusion. We further demonstrate that Omicron BA.1 has an increased efficiency on MT-MMP usage, while an altered efficiency on transmembrane serine protease usage for virus entry compared with that of ancestral SARS-CoV-2. These results reveal additional protease determinants for SARS-CoV-2 infection and enhance our understanding on the biology of coronavirus entry.

## Teaser

Omicron has an increased dependence on MT-MMPs, while a decreased dependence on transmembrane serine protease for virus entry.

## INTRODUCTION

Successful infection of severe acute respiratory syndrome coronavirus 2 (SARS-CoV-2) requires the direct interaction of SARS-CoV-2 spike with its cell surface receptor, angiotensin-converting enzyme 2 (ACE2) (*1*). Similar to other coronaviruses (*2*, *3*), attachment factors, such as heparan sulfate and 78-kDa glucose-regulated protein, facilitate the surface binding of SARS-CoV-2 and promote virus entry (*4*–*6*). Moreover, additional cell surface factors including neuropilin-1 (*7*, *8*), high-density lipoprotein scavenger receptor B type 1 (*9*), CD209/CD299 (*10*), and AXL (*11*) have recently been suggested to play a role in SARS-CoV-2 infection. Apart from the physical interaction between SARS-CoV-2 spike and ACE2 and other cell surface factors, infection of SARS-CoV-2 requires proteolytic cleavage of the viral spike protein with host proteases. The spike proteins of SARS-CoV-2 are first activated by cellular proprotein convertases such as furin. During SARS-CoV-2 infection of target cells, the spike proteins on virus particles are further activated by transmembrane protease serine 2 (TMPRSS2) at the plasma membrane (*1*). In cell types with low or no TMPRSS2 expression, SARS-CoV-2 alternatively enters through endocytic pathways where the spike proteins on virus particles are activated by cathepsin L in the endosomes (*12*–*14*).

Recent evidence suggested that in addition to TMPRSS2, other transmembrane serine proteases including TMPRSS4, TMPRSS11D, and TMPRSS13 can similarly activate SARS-CoV-2 spike at the plasma membrane (*15*–*18*). However, the potential role of alternative transmembrane proteases in facilitating SARS-CoV-2 entry remains incompletely understood. In this study, we reveal that in addition to transmembrane serine proteases, members of the membrane-type matrix metalloproteinase (MT-MMP) and a disintegrin and metalloproteinase (ADAM) families also facilitate the entry of SARS-CoV-2 and other coronaviruses. We show that MT-MMP inhibition significantly limits SARS-CoV-2 replication in Calu3 human lung cells and in the lungs of SARS-CoV-2–infected hamsters, suggesting that MT-MMPs play a physiologically relevant role during SARS-CoV-2 infection. SARS-CoV-2 Omicron BA.1 emerged in late 2021 and quickly replaced Delta as the dominant circulating SARS-CoV-2 variant (*19*). As of September 2022, Omicron sublineages such as BA.4/5 remain as the predominant SARS-CoV-2 variants. These variants contain a large number of mutations, which contributed to their virological features including robust immunoevasiveness, altered TMPRSS2 usage and entry preference, and attenuated pathogenicity (*20*–*24*). Here, we further demonstrate that SARS-CoV-2 Omicron BA.1 (B.1.1.529.1) has an increased efficiency on MT-MMP usage in comparison to that of the ancestral SARS-CoV-2. Overall, our study reveals additional protease determinants for SARS-CoV-2 infection and contributes to our understanding on the biology of coronavirus entry.

## RESULTS

### MT-MMPs and ADAMs facilitate SARS-CoV-2 entry

**Fig. 1.**
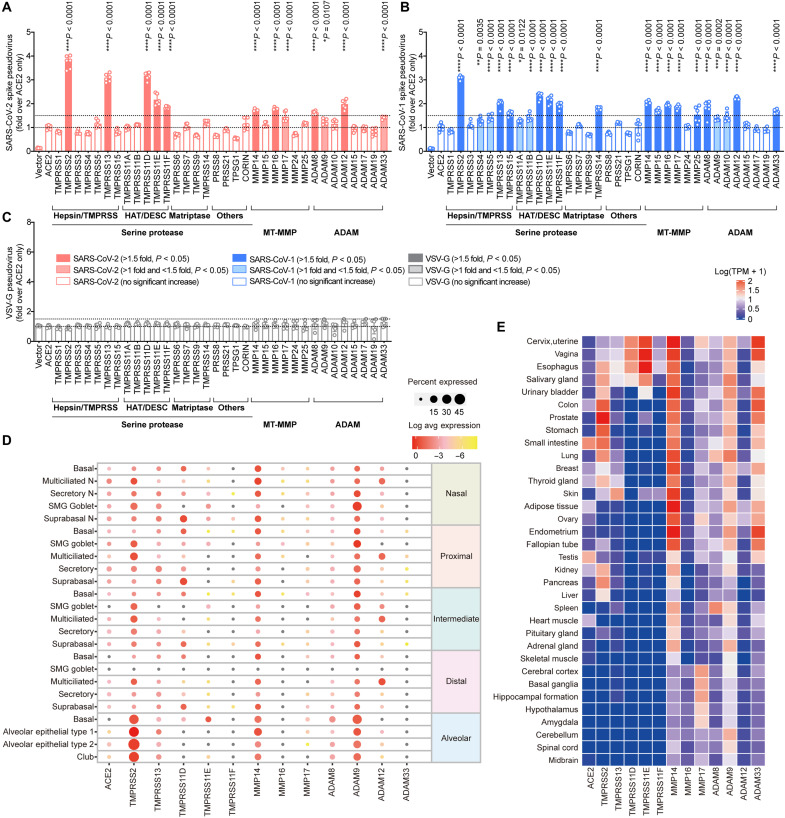
MT-MMPs and ADAMs facilitate SARS-CoV-2 entry. (**A** to **C**) Pseudovirus entry screening. 293T cells were cotransfected with ACE2 and the indicated transmembrane serine protease, MT-MMPs, or ADAMs and then challenged by (A) SARS-CoV-2-S (Wuhan-Hu-1 strain), (B) SARS-CoV-1-S, or (C) VSV-G pseudoviruses at 24 hours after transfection. Pseudoviruses entry was quantified by measuring the luciferase signal of the cell lysates at 24 hours after transduction (*n* = 6). The fold change was normalized with the ACE2 transfection group. (**D**) Single-cell RNA sequencing (scRNA-seq) analysis for the expression of identified transmembrane serine protease, MT-MMPs, or ADAMs in the various cell types of human upper and lower respiratory tract, including the nasal, proximal, intermediate, distal, and alveolar epithelium. (**E**) Bulk RNA sequencing analysis for the expression of the identified transmembrane serine protease, MT-MMPs, or ADAMs in different human organs, reported as log-transformed transcript per million mapped reads [log_10_(TPM + 1)]. The experiments in (A) to (C) were repeated three times independently with similar results. Data represented means and SDs from the indicated number of biological repeats. Statistical significance between groups was determined with one-way analysis of variance (ANOVA). *P < 0.05, **P < 0.01, ***P < 0.001, and ****P < 0.0001.

Proteases anchored on the cell surface, including membrane-anchored serine proteases, MT-MMPs, and ADAMs, mediate the pericellular activation of protein precursors. The role of membrane-anchored serine proteases on proteolytic activation of coronavirus spike for membrane fusion and successful infection of host cells has been well established (*25*–*27*). While ADAM10 and ADAM17 were recently reported to promote SARS-CoV-2 infection (*28*, *29*), whether or not MT-MMPs and other ADAMs are involved in coronavirus entry remains incompletely understood. To this end, we first evaluated MT-MMPs (MMP14, MMP15, MMP16, MMP17, MMP24, and MMP25) (*30*) and a list of ADAMs with known lung or broad tissue distribution (ADAM8, ADAM9, ADAM10, ADAM12, ADAM15, ADAM17, ADAM19, and ADAM33) (*31*, *32*) for their capacities to mediate SARS-CoV-2 entry using vesicular stomatitis virus (VSV)–based SARS-CoV-2-spike (S) pseudoviruses (fig. S1). Our results demonstrated that 5 of the 20 evaluated serine proteases, including TMPRSS2, TMPRSS13, TMPRSS11D, TMPRSS11E, and TMPRSS11F, significantly facilitated SARS-CoV-2 entry, which is in keeping with recent reports (*15*–*18*). Our data further revealed that three of the six MT-MMPs (MMP14, MMP16, and MMP17) and four of the eight ADAMs (ADAM8, ADAM9, ADAM12, and ADAM33) also mediated SARS-CoV-2-S pseudovirus entry, albeit at a lower efficiency when compared to serine proteases (Fig. 1A), suggesting that additional membrane proteases other than serine proteases can also augment SARS-CoV-2 infection. In parallel, we evaluated the capacity of MT-MMPs and ADAMs in mediating SARS-CoV-1 entry. Under the same experimental setting, five of the six MT-MMPs and five of the eight ADAMs facilitated SARS-CoV-1-S pseudovirus entry (Fig. 1B). SARS-CoV-2 has been postulated to originate from bats and jumped to humans through pangolins or other intermediate host animals (*33*, *34*). The spike protein of SARS-CoV-2 is highly similar to that of the pangolin coronavirus (PCoV)–GX–P5L with 92.6% amino acid sequence identity (*34*). Our data demonstrated that MT-MMPs and ADAMs facilitated the entry of PCoV-GX-P5L-S pseudovirus with a pattern similar to that of SARS-CoV-2-S and SARS-CoV-1-S pseudovirus (fig. S2). As a control, none of the evaluated membrane serine proteases, MT-MMPs, and ADAMs enhanced the entry of VSV glycoprotein (VSV-G) pseudovirus (Fig. 1C). Together, these findings reveal that MT-MMPs and ADAMs can augment the entry of SARS-CoV-2 and other coronaviruses.

To evaluate the potential physiological relevance of the identified MT-MMPs and ADAMs in the natural course of SARS-CoV-2 infection of the respiratory tract, we analyzed the human airway (*35*) and human lung (*36*) single-cell RNA sequencing (scRNA-seq) datasets for the expression of TMPRSS2, TMPRSS13, TMPRSS11D, TMPRSS11E, TMPRSS11F, MMP14, MMP16, MMP17, ADAM8, ADAM9, ADAM12, and ADAM33, which significantly augment SARS-CoV-2-S pseudovirus entry. Our analysis revealed that TMPRSS2 and other transmembrane serine proteases were well expressed in the human respiratory tract (Fig. 1D). Similarly, the evaluated MT-MMPs and ADAMs were readily detected in multiple cell types of the human respiratory tract, with MMP14 and ADAM9 demonstrating the most robust expression (Fig. 1D). MMP14, MMP16, MMP17, ADAM8, ADAM9, and ADAM12 were also detected in the nasal epithelial cells, at comparable levels as the transmembrane serine proteases, which highlighted their potential roles in SARS-CoV-2 infection at the human upper respiratory tract (Fig. 1D). In addition to the respiratory tract, SARS-CoV-2 also infects multiple extrapulmonary tissues (*37*, *38*). Our analyses revealed that some of the evaluated MT-MMPs and ADAMs, including MMP14, ADAM9, and ADAM33, have a broad extrapulmonary tissue tropism, including in tissues with low or no TMPRSS2 expression (Fig. 1E). Besides the RNA expression profiles, we similarly demonstrated that the identified MT-MMPs and ADAMs were expressed in physiological-relevant tissues on the protein level (fig. S3). Overall, the expression analyses suggest that the identified MT-MMPs and ADAMs are expressed in physiologically important sites that are relevant to SARS-CoV-2 infection, including the human respiratory tract and extrapulmonary tissues.

### MT-MMPs and ADAMs play functional roles in SARS-CoV-2 replication

**Fig. 2.**
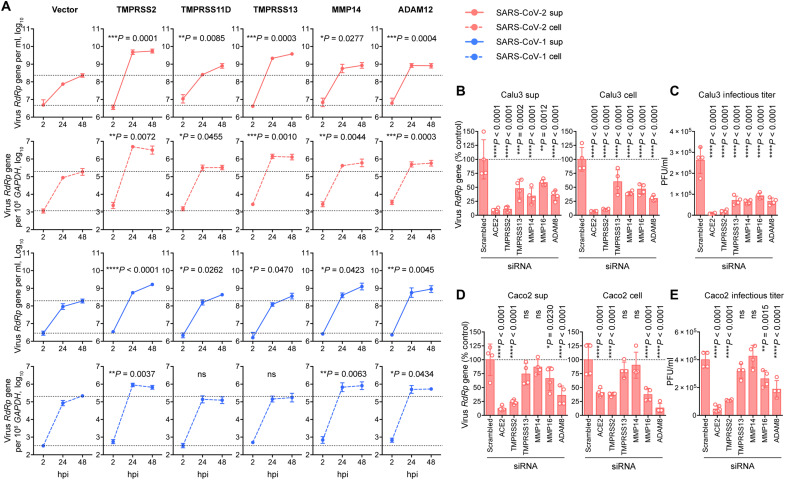
MT-MMPs and ADAMs play functional roles in SARS-CoV-2 replication. (**A**) Authentic virus replication in 293T cells cotransfected with hACE2 and selected proteases. 293T cells were cotransfected with ACE2 and selected proteases and infected with authentic SARS-CoV-2 wild-type (WT) or SARS-CoV-1 viruses at 0.05 MOI for 2 hours at 37°C. Cell lysates and supernatants were harvested at 2, 24, and 48 hpi. Viral RNA-dependent RNA polymerase (RdRp) gene copy was determined with quantitative reverse transcription polymerase chain reaction (qRT-PCR) (*n* = 6). The amount of viral RdRp gene produced between 2 and 48 hpi was calculated and compared with the area under the curve analysis. (**B** to **E**) siRNA knockdown of selected proteases in Calu3 and Caco2 cells. Calu3 and Caco2 cells were treated with siRNA against the indicated transmembrane serine protease, MT-MMPs, or ADAMs. Additional cells were treated with ACE2 or scrambled siRNA as positive and negative controls, respectively. The siRNA-treated cells were challenged with SARS-CoV-2 WT at 0.5 MOI for 2 hours at 37°C. Cell lysates and supernatants were harvested at 24 hpi for qRT-PCR analysis and plaque assay titration (*n* = 4). The experiments were repeated three times independently with similar results. Data represented means and SDs from the indicated number of biological repeats. Statistical significance between groups in (A) and (B) to (E) was determined with area under the curve analysis and one way-ANOVA, respectively. *P < 0.05, **P < 0.01, ***P < 0.001, and ****P < 0.0001. ns, not significant.

With pseudovirus entry assays, we identified a number of MT-MMPs and ADAMs that could facilitate the entry of SARS-CoV-2 pseudovirus. We next sought to validate their roles in facilitating SARS-CoV-2 infection by using authentic virus infection. Within the evaluated MT-MMPs and ADAMs, MMP14 and ADAM12 were among the top MT-MMPs/ADAMs that most efficiently facilitated SARS-CoV-2-S pseudovirus entry (Fig. 1A). We overexpressed them together with human ACE2 in 293T cells followed by SARS-CoV-2 infection and quantified virus replication over time. In parallel, TMPRSS2-, TMPRSS11D-, or TMPRSS13-transfected cells were similarly infected with SARS-CoV-2 as positive control groups for comparison. On the basis of the area under the curve calculation of virus generated between 2 and 48 hours postinfection (hpi), our results demonstrated that exogenous expression of MMP14 and ADAM12 both significantly promoted SARS-CoV-2 replication in comparison to the vector-transfected control (Fig. 2A). Specifically, MMP14 and ADAM12 expression increased the viral gene copy in the supernatant of SARS-CoV-2–infected cells by 5.4- (*P* = 0.0277) and 6.9-fold (*P* = 0.004), respectively (Fig. 2A). In agreement with the SARS-CoV-1-S pseudovirus entry results, MMP14 and ADAM12 similarly facilitated the replication of SARS-CoV-1, which was evidenced by the significant increase in virus replication detected from both supernatant and cell lysate samples (Fig. 2A). Next, we further evaluated the physiological relevance of the identified MT-MMPs and ADAMs with small interfering RNA (siRNA)–mediated gene depletion in human lung epithelial (Calu3) and human intestinal epithelial (Caco2) cells. We selected MMP14, MMP16, and ADAM8 as candidates for evaluation because they are expressed in both Calu3 and Caco2 cells (figs. S4 and S5) while efficiently facilitating SARS-CoV-2 pseudovirus entry (Fig. 1A). In addition to MMP14, MMP16, and ADAM8, we similarly depleted TMPRSS2 and TMPRSS13 with siRNA as positive controls (Fig. 2B). The siRNA-treated cells were infected with SARS-CoV-2 and harvested at 24 hpi. In keeping with a recent report (*17*), our results demonstrated that depletion of either TMPRSS2 or TMPRSS13 in Calu3 cells significantly decreased SARS-CoV-2 replication (Fig. 2B). Despite to a smaller extent compared to TMPRSS2, depletion of MMP14, MMP16, and ADAM8 significantly reduced SARS-CoV-2 viral genome copies and lowered the infectious virus titers (MMP14: 4.2-fold, *P* < 0.0001; MMP16: 2.8-fold, *P* < 0.0001; DAM8: 4.0-fold, *P* < 0.0001) in Calu3 cells when compared to the scrambled siRNA–treated cells (Fig. 2, B and C). We noticed a cell type–dependent phenomenon on protease usage for SARS-CoV-2 replication as depletion of TMPRSS2, MMP16, and ADAM8, but not TMPRSS13 and MMP14, decreased SARS-CoV-2 replication in Caco2 cells (Fig. 2, D and E).

**Fig. 3.**
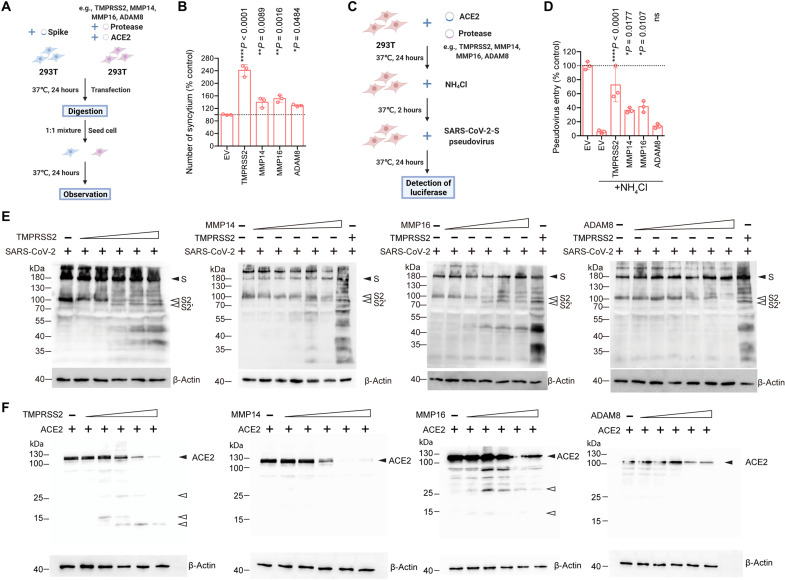
Mechanistic investigation on how MMP and ADAM facilitate SARS-CoV-2 replication. (**A**) Schematic of the cell-cell fusion assay. (**B**) SARS-CoV-2-S (Wuhan-Hu-1)–transfected 293T cells were cocultured with 293T cells cotransfected with ACE2 and the indicated proteases. The number of syncytium was counted under a microscope and normalized with the control group (*n* = 3). (**C**) Schematic of the SARS-CoV-2 pseudovirus entry assay with NH_4_Cl treatment. (**D**) 293T cells cotransfected with ACE2 and the indicated proteases were pretreated with NH_4_Cl, followed by SARS-CoV-2-S (Wuhan-Hu-1) pseudovirus transduction. Pseudoviruses entry was quantified at 24 hours after transduction (*n* = 3). The result was normalized with the control group (293T cotransfected with ACE2 and empty plasmid without NH_4_Cl pretreatment). (**E**) Western blot of SARS-CoV-2 spike cleavage by MMPs and ADAMs. Plasmids of identified proteases and SARS-CoV-2 spike were cotransfected in 293T cells. Cell lysates at 24 hours after transfection were harvested for Western blot detection of SARS-CoV-2 spike cleavage. SARS-CoV-2 spike from the Wuhan-Hu-1 strain was used in (E). (**F**) Western blot of human ACE2 cleavage by protease. Identified proteases and human ACE2 were coexpressed in 293T cells. Cell lysates at 24 hours after transfection were harvested for Western blot detection of hACE2 cleavage. The experiments were repeated three times independently with similar results. Data in (B) and (D) represented means and SDs from the indicated number of biological repeats. Statistical significance between groups in (B) and (D) was determined with one way-ANOVA. *P < 0.05, **P < 0.01, and ****P < 0.0001. EV, empty vector.

To obtain mechanistic insights on how MT-MMPs and ADAMs augment SARS-CoV-2 entry, we first evaluated the role of MMP14, MMP16, and ADAM8 in fusion assays (Fig. 3A). Our results demonstrated that MMP14, MMP16, and ADAM8 expression significantly increased SARS-CoV-2 spike–ACE2–mediated cell-cell fusion, which is in line with their role in facilitating SARS-CoV-2 entry (Fig. 3B). Next, to delineate whether MMP14, MMP16, and ADAM8 act to promote virus entry at the plasma membrane or in the endosomes, we performed SARS-CoV-2 pseudovirus entry assays in the presence of ammonium chloride (NH_4_Cl), which abolishes endosomal entry (Fig. 3C) (*1*). Our results demonstrated that MMP14 and MMP16 rescued NH_4_Cl-inhibited virus entry from 5.5 to 36.4% (*P* = 0.0177) and 41.6% (*P* = 0.0107), respectively, relative to the control (Fig. 3D). In contrast, ADAM8 did not significantly rescue virus entry from NH_4_Cl-mediated entry inhibition [5.5 to 13.8%, *P* = ns (not significant)] (Fig. 3D). Thus, these results suggested that MMP14- and MMP16-facilitated SARS-CoV-2 entry did not rely on the endocytic machinery and might promote virus entry at the plasma membrane, while ADAM8 predominantly promoted SARS-CoV-2 entry through the endosomal entry pathway.

Next, we asked whether the identified MT-MMPs and ADAMs could mediate viral spike cleavage. To this end, we cotransfected SARS-CoV-2 spike together with MMP14, MMP16, or ADAM8 and evaluated spike cleavage with Western blots, with TMPRSS2 included as a positive control. Our results demonstrated that MMP14 and MMP16 could mediate SARS-CoV-2 spike cleavage. Similar to spike cleavage mediated by TMPRSS2, we observed increased cleavage at the S2′ site, whereas no increase in S_1_/S_2_ cleavage was observed upon MMP14/16 expression (Fig. 3E). Apart from MMP14 and MMP16, additional bands of SARS-CoV-2 spike slightly above 100 kDa were detected from cells with ADAM8/spike overexpression (Fig. 3E). Since TMPRSS2 also cleaves the host ACE2, which is required for the entry of SARS-CoV-1 (*26*, *39*) and SARS-CoV-2 (fig. S6), we asked whether MT-MMPs and ADAMs could similarly cleave ACE2. To address this possibility, we cotransfected ACE2 together with MMP14, MMP16, ADAM8, or TMPRSS2 and evaluated ACE2 cleavage with Western blots. Our data showed that among the evaluated MT-MMPs and ADAMs, only MMP16 cleaved ACE2. The ACE2 cleavage pattern mediated by MMP16 was not completely the same when compared to that of TMPRSS2-mediated ACE2 cleavage (Fig. 3F). We also noticed that the capacity of a certain protease to cleave SARS-CoV-2 spike and ACE2 did not necessarily translate to its capacity in facilitating SARS-CoV-2 entry, since TMPRSS1, which cleaved both SARS-CoV-2 spike and ACE2 (fig. S7), did not facilitate SARS-CoV-2 entry according to our pseudovirus entry assay (Fig. 1A). Collectively, our results demonstrate the role of MT-MMPs and ADAMs in facilitating SARS-CoV-2 entry. In particular, we identify MT-MMP as a class of protease that can cleave SARS-CoV-2 spike for virus entry at the cell surface in addition to serine proteases.

### Pan-MMP inhibitors reduce SARS-CoV-2 replication in vitro

**Fig. 4.**
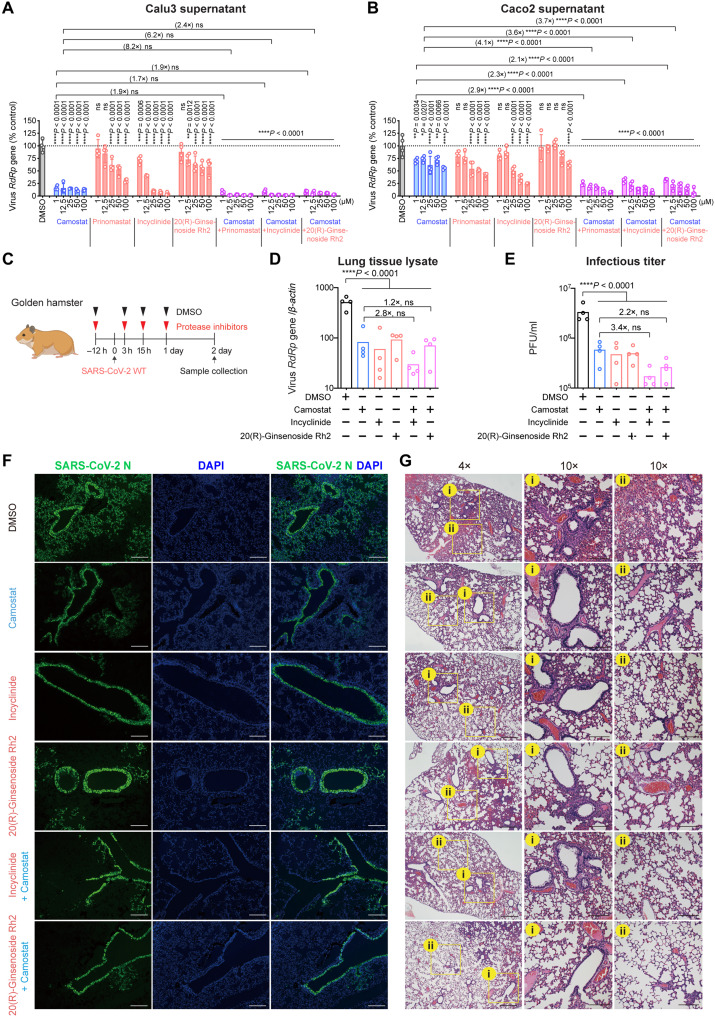
Pan-MMP inhibitors reduce WT SARS-CoV-2 replication in vitro and in vivo. (**A** and **B**) Calu3 and Caco2 cells were treated with DMSO, pan-serine protease inhibitor (camostat), pan-MMP inhibitors (prinomastat, incyclinide, and 20(R)-ginsenoside Rh2) or at the indicated combination for 2 hours at 37°C. The pretreated cells were challenged with SARS-CoV-2 WT. Supernatant samples were harvested at 24 hpi for qRT-PCR analysis (*n* = 4). (**C**) Schematic the pan-MMP inhibitor experiment in golden hamsters. (**D** and **E**) Golden hamster lung samples were harvested on day 2 after SARS-CoV-2 WT challenge and homogenized for qRT-PCR analysis and plaque assay titration (*n* = 4). (**F**) Representative immunofluorescence images of infected hamster lungs with or without treatments. SARS-CoV-2 nucleocapsid (N) protein was identified with a rabbit anti–SARS-CoV-2-N immune serum (green), and nuclei was identified with 4′,6-diamidino-2-phenylindole (DAPI) stain (blue). (**G**) Representative hematoxylin and eosin images of infected hamster lungs with or without treatments. Representative regions of (i) bronchiole epithelium and (ii) alveolar space were shown. Scale bars in (F) and (G) represented 500 or 200 μm for 4-fold or 10-fold magnifications of the objective, respectively, with ×10 magnification at the eyepiece. The experiments in (A) and (B) and (D) to (G) were repeated three times and two times independently with similar results, respectively. Data represented means and SDs from the indicated number of biological repeats. Statistical significance between groups in (A) and (B), and (D) and (E) was determined with two-way and one-way ANOVA, respectively. *P < 0.05, **P < 0.01, ***P < 0.001, and ****P < 0.0001.

Given the important role of MT-MMPs in mediating SARS-CoV-2 entry, we next asked whether inhibiting MT-MMPs could limit SARS-CoV-2 replication. To address this question, we treated SARS-CoV-2–infected Calu3 cells with pan-MMP inhibitors, including prinomastat, incyclinide, or 20(R)-ginsenoside Rh2, and quantified virus replication at 24 hpi. In parallel, SARS-CoV-2–infected Calu3 cells were similarly treated with the pan-transmembrane serine protease inhibitor, camostat, as a positive control. Our results demonstrated a potent inhibitory effect of camostat on SARS-CoV-2 replication, in keeping with previous reports (*1*, *16*). At the same time, all three evaluated pan-MMP inhibitors inhibited SARS-CoV-2 replication in Calu3 cells in a dose-dependent manner (Fig. 4A and figs. S8 and S9). Our results showed that the pan-MMP inhibitors and camostat functioned in an additive manner in suppressing SARS-CoV-2 replication. For instance, 1 μM camostat reduced SARS-CoV-2 RNA-dependent RNA polymerase (RdRp) gene copy in the Calu3 supernatants to 16.2% comparing to the dimethyl sulfoxide (DMSO)–treated cells (Fig. 4A). At the same time, 1 μM camostat added together with 1 μM prinomastat, incyclinide, or 20(R)-ginsenoside Rh2 reduced SARS-CoV-2 RdRp gene copy in the Calu3 supernatants to 8.5, 9.3, or 8.7%, respectively, comparing to the DMSO-treated cells. This level of virus inhibition was not achieved by treating the cells with camostat only, even when treated at 100 μM, which reduced virus replication to 12.0% of the DMSO-treated cells (Fig. 4A). In parallel, we evaluated the inhibitory effect of pan-MMP inhibitors on SARS-CoV-2 replication in Caco2 cells. In line with the findings in Calu3 cells, pan-MMP inhibitors similarly limited the replication of SARS-CoV-2 in Caco2 cells. The additive effect of camostat and pan-MMP inhibitors was highly appreciable in Caco2 cells. Specifically, 1 μM camostat reduced SARS-CoV-2 RdRp gene copy in the Caco2 supernatants to 70.1% of the DMSO-treated cells. Together with 1 μM camostat and 1 μM prinomastat, incyclinide, or 20(R)-ginsenoside Rh2, SARS-CoV-2 RdRp gene copy in Caco2 supernatants was further reduced to 23.9, 30.5, or 32.7%, respectively, comparing to the DMSO-treated cells (Fig. 4B). Similar patterns of virus inhibition were also observed in the Calu3 and Caco2 cell lysate samples (fig. S9). Overall, these results demonstrate that the pan-MMP inhibitors suppress SARS-CoV-2 replication in vitro. When used in conjunction with camostat, the pan-MMP inhibitors and the pan-serine protease inhibitor function in an additive manner to efficiently inhibit SARS-CoV-2 replication.

### Pan-MMP inhibitors limit SARS-CoV-2 replication and pathogenesis in golden Syrian hamsters

To further evaluate the physiological relevance of MT-MMP inhibition in vivo, we pretreated golden hamsters with incyclinide or 20(R)-ginsenoside Rh2, with or without camostat, through the intranasal route. At 12 hours after pretreatment, we intranasally inoculated the hamsters with SARS-CoV-2, followed by additional inhibitor treatment at 3 hours, 15 hours, and day 1 after virus inoculation, and sacrificed the hamsters at day 2 after virus inoculation (Fig. 4C and fig. S8B). Our results suggested that incyclinide and 20(R)-ginsenoside Rh2 significantly reduced SARS-CoV-2 replication in the hamster lungs, which was evidenced by the significantly lowered viral RdRp gene copy (Fig. 4D) and infectious virus titers (Fig. 4E) upon treatment. In line with the in vitro findings, the combined camostat/incyclinide and camostat/20(R)-ginsenoside Rh2 treatments suppressed SARS-CoV-2 infectious titer at 3.4- and 2.2-fold more efficiently than the camostat single treatment, respectively, albeit the difference was not statistically significant (Fig. 4E). We next analyzed the expression of SARS-CoV-2 nucleocapsid (N) protein in the harvested hamster lungs. We detected abundant viral N expression in the bronchiole epithelial cells and in the alveolar space in DMSO-treated hamster lungs. In contrast, in incyclinide or 20(R)-ginsenoside Rh2–treated hamsters, viral N gene expression was more confined to the bronchiole epithelium, suggesting that pan-MMP inhibitors limited the dissemination of SARS-CoV-2 in hamster lungs (Fig. 4F). In parallel, histopathological examination showed severe bronchiolar epithelial desquamation with extensive peribronchiolar mononuclear cell infiltration at the bronchiolar epithelium (Fig. 4G, DMSO, i), while alveolar congestion, infiltration, and hemorrhage were detected in the alveolar space (Fig. 4G, DMSO, ii) in the lung of SARS-CoV-2–infected hamsters receiving mock treatments. These pathological findings are consistent with what we have previously reported in SARS-CoV-2–infected hamsters (*40*). In contrast, in incyclinide or 20(R)-ginsenoside Rh2–treated hamsters, the lung damage was markedly improved as we observed a mild degree of bronchiolar epithelium damage as well as milder and more localized infiltrations in the alveolar space (Fig. 4G). In both of the immunofluorescence and histology studies, the pan-MMP and pan-transmembrane serine protease inhibitors independently ameliorated lung infection and lung pathology. We did not observe further improved virus clearance or lung pathology beyond the independent treatments with the camostat/incyclinide or camostat/20(R)-ginsenoside Rh2 combined treatment (Fig. 4, F and G, and fig. S10), potentially due to sensitivity limitations of these imaging-based assays. Nevertheless, our results reveal that the pan-MMP inhibitors reduce SARS-CoV-2 replication and ameliorate viral pathogenesis in vivo.

### Altered host protease usage by SARS-CoV-2 Omicron BA.1

**Fig. 5.**
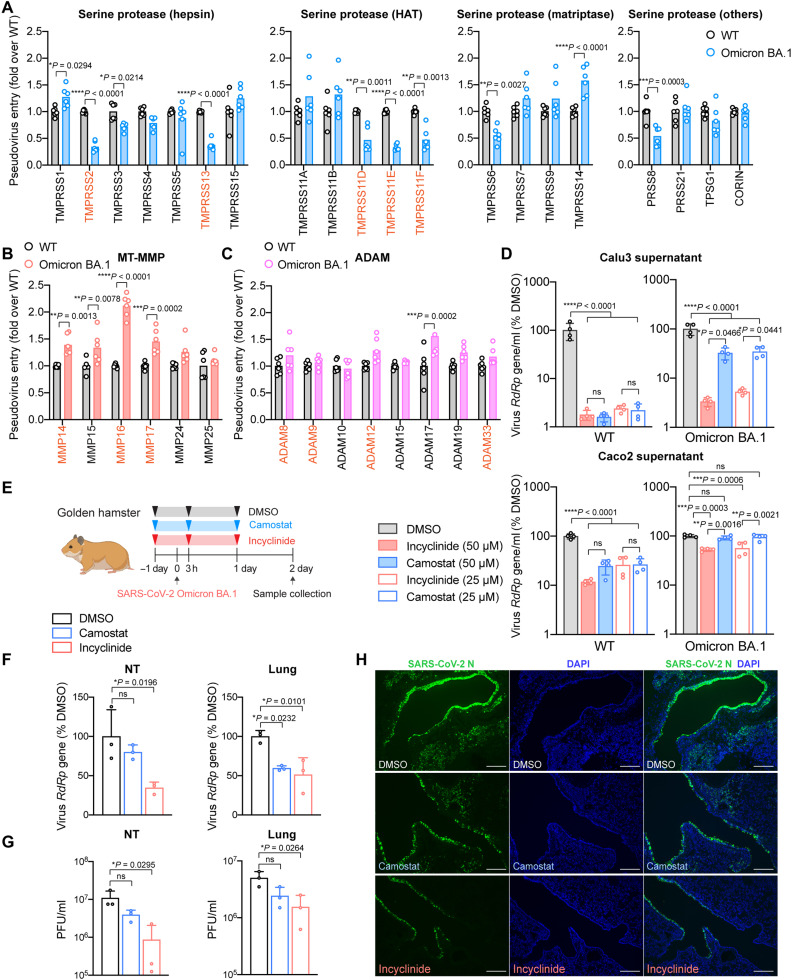
Altered host protease usage for SARS-CoV-2 Omicron BA.1. (**A** to **C**) 293T cells were cotransfected with hACE2 and the indicated proteases, followed by transduction with SARS-CoV-2-S (Wuhan-Hu-1) or Omicron BA.1-S pseudoviruses. Pseudoviruses entry was determined by quantifying the luciferase signal of the cell lysates at 24 hours after transduction (*n* = 6). (**D**) Calu3 and Caco2 cells were pretreated with DMSO, pan-serine protease inhibitor (camostat), or pan-MMP inhibitor (incyclinide) for 2 hours. The pretreated cells were challenged with either SARS-CoV-2 WT or Omicron BA.1 at 0.5 multiplicity of infection (MOI). Infected cells were maintained for 48 hours in the presence of inhibitors. Supernatant samples were harvested at 48 hpi for qRT-PCR analysis (*n* = 4). (**E**) Schematic of pan-MMP inhibitor (incyclinide) against Omicron BA.1 in golden hamsters. (**F** and **G**) Golden hamster lung and NT samples were harvested on day 2 after Omicron BA.1 challenge and homogenized for qRT-PCR analysis and plaque assay titration (*n* = 3). (**H**) Representative immunofluorescence images of infected hamster lungs with or without treatments. SARS-CoV-2 N protein was identified with a rabbit anti–SARS-CoV-2-N immune serum (green), and nuclei were identified with DAPI stain (blue). Scale bars, 200 μm for 100-fold magnifications. The experiments in (A) to (D) were repeated three times independently with similar results. Animal experiments in (F) to (H) were repeated twice independently with similar results. Data represented means and SDs from the indicated number of biological repeats. Statistical significance between groups in (A-C) and (D-G) was determined with two-way and one-way ANOVA, respectively. *P < 0.05, **P < 0.01, ***P < 0.001, and ****P < 0.0001.

SARS-CoV-2 Omicron BA.1 (B.1.1.529.1) emerged in November 2021 and replaced Delta as the dominant SARS-CoV-2 variant. We and others recently demonstrated that Omicron BA.1 is less efficient in spike cleavage at the S_1_/S_2_ cleavage site, resulting in reduced TMPRSS2 usage and substantially attenuated pathogenicity in infected animals (*20*, *22*, *41*, *42*). By comparing the entry of SARS-CoV-2 wild-type (WT) and Omicron BA.1 pseudoviruses, we demonstrated that Omicron BA.1 was less efficient in using the transmembrane serine proteases that could facilitate SARS-CoV-2 WT entry, which included TMPRSS2, TMPRSS13, TMPRSS11D, TMPRSS11E, and TMPRSS11F (Fig. 5A). Nonetheless, among the evaluated serine proteases, Omicron BA.1 used TMPRSS1 and TMPRSS14 at an increased efficiency when compared to WT (Fig. 5A). Notably, Omicron BA.1 demonstrated a higher efficiency in using all three MT-MMPs that could facilitate SARS-CoV-2 WT entry (MMP14, MMP16, and MMP17) (Fig. 5B). In addition, SARS-CoV-2 WT and Omicron BA.1 used ADAMs at comparable efficiencies with the exception of ADAM17 (Fig. 5C). Together, these results indicate that Omicron BA.1 is less efficient in using serine proteases that could facilitate SARS-CoV-2 WT entry, including TMPRSS2, TMPRSS13, TMPRSS11D, TMPRSS11E, and TMPRSS11F. In contrast, Omicron BA.1 is more potent in using MT-MMPs when compared to SARS-CoV-2 WT. To further evaluate the role of serine proteases and MT-MMPs during SARS-CoV-2 WT and Omicron BA.1 infection, we treated SARS-CoV-2 WT or Omicron BA.1–infected Calu3 and Caco2 cells with camostat or incyclinide, followed by quantification of virus replication in the culture supernatant at 48 hpi. Our results demonstrated that while both camostat and incyclinide potently inhibited the replication of SARS-CoV-2 WT in Calu3 cells, the replication of Omicron BA.1 was more readily inhibited by incyclinide than camostat. At 25 μM, incyclinide and camostat reduced Omicron BA.1 replication to 5.3% and 34.5% (*P* = 0.0441 comparing incyclinide and camostat) than that of mock treatment at 48 hpi, respectively (Fig. 5D). In Caco2 cells, while both camostat and incyclinide efficiently inhibited the replication of SARS-CoV-2 WT, the replication of Omicron BA.1 was only significantly inhibited by incyclinide (25 μM: 56.3% of mock, *P* = 0.0006) but not camostat (25 μM: 94.3% of mock, *P* = ns) (Fig. 5D). In addition, we evaluated the role of pan-MMP inhibition on WT and Omicron BA.1 infection in 293T-ACE2 (fig. S11A) and A549-ACE2 cells (fig. S11B), which have low/no TMPRSS2 expression. Our results demonstrated that E64D was more potent than incyclinide in inhibiting the replication of WT and Omicron BA.1 in both cells. Meanwhile, both E64D and incyclinide inhibited Omicron BA.1 at a higher efficiency in these cells when compared with WT. Last, we evaluated the effect of camostat and incyclinide in inhibiting Omicron BA.1 replication in golden hamsters (Fig. 5E). Our results demonstrated that Omicron BA.1 replication in nasal turbinates (NTs) and lungs in the infected hamsters were more efficiently inhibited by incyclinide in comparison to camostat as evidenced by the more appreciable decrease in both virus RdRp gene copy (Fig. 5F) and infectious virus titer (Fig. 5G). In the lung samples, camostat reduced Omicron BA.1 infectious titer by 51.7% (*P* = ns), while incyclinide reduced Omicron BA.1 infectious titer by 69.2% (*P* = 0.0264) (Fig. 5G). In keeping with these observations, immunofluorescence staining of viral N protein similarly detected less viral antigen in the incyclinide-treated hamster lung samples in comparison to the camostat-treated hamster lung samples (Fig. 5H and fig. S12). Overall, our results reveal that SARS-CoV-2 can use MT-MMPs for virus entry, and SARS-CoV-2 Omicron BA.1 has an increased efficiency on MT-MMP usage than the ancestral SARS-CoV-2.

## DISCUSSION

Despite extensive studies exploring the host cell determinants for SARS-CoV-2 entry (*4*–*11*), the host proteases that facilitate SARS-CoV-2 entry at the target cells have not been comprehensively explored. The requirement of TMPRSS2 on SARS-CoV-2 entry was revealed early in the pandemic (*1*). More recently, the involvement of other transmembrane serine proteases during SARS-CoV-2 entry has been demonstrated for TMPRSS4 (*15*), TMPRSS11D (*16*, *18*), TMPRSS11E (*16*, *18*), TMPRSS11F (*16*), and TMPRSS13 (*16*–*18*). However, the role of host protease beyond transmembrane serine proteases has not been thoroughly investigated for the entry of SARS-CoV-2 or other coronaviruses. Previous study found that inhibition of MMPs and ADAMs markedly decreased both entry and cell-cell fusion of murine coronavirus mouse hepatitis virus, hinting that these metalloproteases should be further investigated for their roles on coronavirus entry (*43*). More recently, the role of ADAM10 and ADAM17 has been demonstrated for SARS-CoV-2 (*28*, *29*). Here, we identified MT-MMPs and additional ADAMs that facilitate the entry of SARS-CoV-2 and other coronaviruses. Among these identified proteases, MT-MMPs are of particular interest as they mediate SARS-CoV-2 spike and ACE2 cleavage, promote spike-mediated fusion, and can facilitate SARS-CoV-2 entry through the plasma membrane entry pathway. pan-MMP inhibitors independently limit SARS-CoV-2 replication in vitro and in vivo and demonstrate an additive effect when administered in conjunction with the pan-serine protease inhibitor, camostat. These results suggest that SARS-CoV-2 entry is orchestrated simultaneously by a repertoire of host proteases in a tissue-dependent manner. Similar to spike cleavage mediated by TMPRSS2, we observed increased cleavage at S2′ upon MMP14 and MMP16 expression, whereas no increase in S_1_/S_2_ cleavage was observed. This finding suggests that MMP14/16 may mediate spike cleavage at or near the S2′ region. Since the prototype cleavage motif of MMPs is different from that of serine proteases, the specific cleavage motif of MMP14/16 on SARS-CoV-2 spike should be further investigated.

SARS-CoV-2 Omicron BA.1 emerged in late 2021 and has quickly replaced Delta as the predominant circulating variant, partly due to its strong capacity of immunoevasion (*21*, *44*). In terms of virological features, Omicron BA.1 spike is inefficiently cleaved by furin, which results in its decreased efficiency in using TMPRSS2 for virus entry in comparison to SARS-CoV-2 WT and previous variants, leading to attenuated pathogenicity (*20*, *22*, *41*, *42*). Here, we show that Omicron BA.1 is less efficient in using all transmembrane serine proteases that could facilitate SARS-CoV-2 WT entry, which included TMPRSS13, TMPRSS11D, TMPRSS11E, and TMPRSS11F, in addition to TMPRSS2. Meanwhile, Omicron BA.1 can use all three MT-MMPs that could facilitate SARS-CoV-2 WT entry (MMP14, MMP16, and MMP17) at significantly higher efficiencies. In addition, the altered Omicron BA.1 protease usage profile also includes the increased usage of TMPRSS1, TMPRSS14, and ADAM17. Now, multiple camostat or other serine protease inhibitor–based strategies are being evaluated in clinical trials as potential treatment strategies against coronavirus disease 2019 (COVID-19). Some have been completed but with suboptimal beneficial outcome (*45*). Considering that SARS-CoV-2 can use multiple host proteases for virus entry and the altered protease usage profile by Omicron BA.1, our study suggests that combined treatments of concurrent serine protease and MMP inhibition should be explored as a treatment strategy against Omicron BA.1 and other Omicron sublineages to achieve the optimal inhibitory effect on virus entry and replication. In addition, it is also important to note that some pan-MMP inhibitors, including incyclinide, are also anti-inflammatory (*46*). In this regard, the use of these inhibitors may provide dual antiviral and anti-inflammation benefits.

Overall, our study revealed additional protease determinants for the infection of SARS-CoV-1, SARS-CoV-2 WT, and SARS-CoV-2 Omicron BA.1.These findings contribute to our understanding on the biology of coronavirus entry.

## MATERIALS AND METHODS

### Viruses and biosafety

SARS-CoV-2 WT HKU-001a (GenBank, accession number: MT230904) and Omicron BA.1 (hCoV-19/Hong_Kong/HKU-211129-001/2021; EPI_ISL_6841980) strains were isolated from the respiratory tract specimens of laboratory-confirmed COVID-19 patients in Hong Kong, as previously described (*22*, *47*). SARS-CoV-1 GZ50 (GenBank, accession number: AY304495) was an archived clinical isolate at the Department of Microbiology, the University of Hong Kong (HKU) (*48*). SARS-CoV-2 and SARS-CoV-1 were cultured using VeroE6-TMPRSS2 and VeroE6 cells, respectively, and titrated by plaque assays. All experiments with infectious SARS-CoV-2 WT, SARS-CoV-2 Omicron BA.1, and SARS-CoV-1 were performed according to the approved standard operating procedures of the biosafety level 3 facility at the Department of Microbiology, HKU.

### Cell cultures

Calu3, Caco2, 293T, Huh7, and VeroE6 were obtained from the American Type Culture Collection. 293T-ACE2 was obtained from GeneCopoeia (Rockville, Maryland, USA). A549-ACE2 was obtained from Invivogen (San Diego, CA, USA). Cells were maintained in Dulbecco’s modified Eagle’s medium (DMEM) (Gibco, Amarillo, Texas, USA) or DMEM/F12 (Gibco) according to the supplier’s instructions (*47*). VeroE6-TMPRSS2 was obtained from the Japanese Collection of Research Bioresources Cell Bank and cultured in DMEM. All cell lines used are routinely tested for mycoplasma and are maintained mycoplasma-free.

### Coronavirus-S pseudoviruses and pseudovirus entry assays

The coronavirus-S pseudovirus used, including SARS-CoV-2-S (WT and Omicron BA.1), SARS-CoV-1-S, and PCoV-GX-P5L-S pseudoviruses, were packaged as described previously (*4*, *49*). Briefly, 293T cells were transfected with different spikes or VSV-G plasmids with Lipofectamine 3000 (Thermo Fisher Scientific, Waltham, MA, USA). At 24 hours after transfection, the cells were transduced with VSV-deltaG-firefly pseudotyped with VSV-G (*49*). At 2 hours after transduction, the cells were washed three times with phosphate-buffered saline (PBS) and cultured in fresh media with anti-VSV-G (8G5F11) antibody (EB0010, Kerafast, Boston, MA, USA). The pseudoviruses were then harvested and titrated with limiting dilutions to determine the 50% tissue culture infectious dose. For pseudovirus entry assays, target cells were inoculated with pseudoviruses for 2 hours and cultured in 1% fetal bovine serum (FBS) media for 24 hours before being washed and lysed for detection of luciferase signal with a luciferase assay system (E1501, Promega, Madison, WI, USA). For pseudovirus entry assays with host protease overexpression, the protease plasmids were obtained from OriGene (Rockville, MD, USA), Sino Biological (Beijing, China), or Addgene (Watertown, Massachusetts, USA). For NH_4_Cl blocking SARS-CoV-2-S pseudovirus entry assays, target cells were pretreated with 50 mM NH_4_Cl for 2 hours at 37°C before inoculating the cells with SARS-CoV-2 pseudovirus. To compare the entry of SARS-CoV-2-WT-S and SARS-CoV-2-Omicron BA.1-S pseudoviruses, the protease expression plasmids and ACE2 plasmid were cotransfected into 293T cells 24 hours before pseudoviruses transduction. WT and Omicron BA.1 pseudovirus entries were quantified at 24 hours after pseudovirus transduction, and the entry of Omicron BA.1-pseudovirus was normalized to that of the WT-pseudovirus.

### scRNA-seq, bulk RNA-seq, and proteomic analysis

The human airway and lung datasets were obtained from public available cohorts. The human airway scRNA dataset was downloaded from https://genomique.eu/cellbrowser/HCA/ (*35*) (European Genome-phenome Archive, accession code EGAS00001004082). The human lung scRNA dataset was downloaded from Synapse (https://synapse.org/#!Synapse:syn21041850) (*36*) under accession code EGAS00001004344. For both datasets, gene expression normalization, log transformation, and visualization were conducted using R package Seurat v.3 (*50*). The “DotPlot” function was applied to visualize gene expression levels across cell types of interest. For bulk RNA-seq data analysis, transcripts per million (TPM)–normalized gene expression data for all the organs listed in the heatmap were retrieved from The Genotype-Tissue Expression Project (Gene Expression Omnibus accession code GSE115828). The data used for the analyses were downloaded from Human Protein Atlas (http://proteinatlas.org) (*51*). R package ggplot2 and pheatmap were used to generate the heatmap visualization of gene expression. Data of protease expression on protein level across normal human tissues were downloaded from the Human Protein Atlas (http://proteinatlas.org) (*51*) and ProteomicsDB (*52*). R package ggplot2 and ComplexHeatmap were used to generate the heatmap visualization of protein levels.

### Golden Syrian hamster model

The use of animals followed all relevant ethical regulations and was approved by the Committee on the Use of Live Animals in Teaching and Research of The University of Hong Kong. Male and female golden Syrian hamsters, aged 6 to 8 weeks old, were obtained from the Chinese University of Hong Kong Laboratory Animal Service Centre through the HKU Centre for Comparative Medicine Research (*53*). To evaluate drug toxicity, hamsters were treated intranasally with camostat (1.25 mg/kg), incyclinide, 20(R)-ginsenoside-Rh2, combination of camostat (0.625 mg/kg) and incyclinide (0.625 mg/kg), combination of camostat (0.625 mg/kg) and 20(R)-ginsenoside-Rh2 (0.625 mg/kg), or DMSO twice per day at an interval of approximately 12 hours for two consecutive days. All intranasal treatment in hamsters was performed under intraperitoneal ketamine (100 mg/kg) and xylazine (10 mg/kg) anesthesia (*54*). Health status and body weight were monitored daily for the toxicity test. To evaluate the antiviral effects of the inhibitors against WT SARS-CoV-2, hamsters were pretreated with one dose of inhibitors 12 hours before virus inoculation intranasally. Next, the hamsters were inoculated with 3 × 10^3^ plaque-forming units (PFU) WT SARS-CoV-2 prediluted in 50 μl of PBS intranasally. The infected hamsters were treated with the inhibitors intranasally at 3 hpi, 15 hpi, and day 1 after infection, respectively. To investigate the antiviral effects of the inhibitors against Omicron BA.1, hamsters were intranasally pretreated with one dose of inhibitors (1.25 mg/kg) or DMSO at 24 hours before virus inoculation, followed by intranasal virus inoculation with 2 × 10^4^ PFU Omicron BA.1 prediluted in 50 μl of PBS. After inoculation, the Omicron BA.1–infected hamsters were treated with inhibitors intranasally at 3 hpi and day 1 after infection, respectively. The drugs in both experiments were dissolved in DMSO and diluted in a solvent composed of 3% ethanol, 7% Tween 80, 0.81% NaCl, and autoclaved double-distilled water. The health status and body weight of hamsters were monitored on a daily basis until the animal was sacrificed or euthanized because of reaching the humane end point of the experiment. Hamsters were sacrificed on day 2 after infection, and lung tissues were harvested for immunofluorescence staining, histopathology examination, quantitative reverse transcription polymerase chain reaction (qRT-PCR), or plaque assays, as we previously described (*40*). In the Omicron BA.1–infected hamsters, NT tissues were also harvested for qRT-PCR and plaque assay analysis, as we previously described (*40*).

### Immunofluorescence staining

Immunofluorescence staining was performed as we previously described (*55*, *56*). Briefly, infected hamster lungs were fixed for 24 hours in 10% formalin. The fixed samples were washed with 70% ethanol and embedded in paraffin by TP1020 Leica semienclosed bench top tissue processor (Leica biosystems, Buffalo Grove, IL, USA) and sectioned with microtome (Thermo Fisher Scientific). Sectioned samples were dewaxed by serially diluted xylene, ethanol, and double-distilled water in sequence. To retrieve the antigens, the sectioned samples were boiled with antigen unmasking solution (H-3300, Vector Laboratories) at 85°C for 90 s, followed by Sudan black B and 1% bovine serum albumin blocking for 30 min, respectively. The in-house rabbit anti–SARS-CoV-2-N immune serum was used as the primary antibodies by incubation at 4°C overnight. The secondary antibody, goat anti-rabbit immunoglobulin G (H + L) fluorescein isothiocyanate (65-6111), was purchased from Thermo Fisher Scientific. The antifade mounting medium with 4′,6-diamidino-2-phenylindole (DAPI; H-1200, Vector Laboratories, Burlingame, CA, USA) was used for mounting and DAPI staining. Images were taken with the Olympus BX53 fluorescence microscope (Olympus Life Science, Tokyo, Japan). Quantification of the immunofluorescence signal was done with ImageJ.

### Histopathology examination

Sectioned hamster lung samples were dewaxed and stained with Gill’s hematoxylin and eosin Y (Thermo Fisher Scientific) to examine the severity of lung tissue damage, as we previously described (*57*, *58*). The histology findings made in our current study represent an unbiased description of the pathological damage in the hamster lung tissues examined, and the results have been validated by an experienced pathologist in a blinded manner.

### RNA extraction and real-time qRT-PCR

RNA extraction and real-time qRT-PCR were performed as previously described with slight modifications (*38*). The QIAsymphony RNA Kit (931636, Qiagen, Germantown Road Germantown, MD, USA) was used to extract RNA of cell lysate and supernatant samples. RNA from hamster lung samples was extracted with the RNeasy Mini kit (74106, Qiagen). qRT-PCR was performed using the QuantiNova SYBR Green RT-PCR Kit (208154, Qiagen) or the QuantiNova Probe RT-PCR Kit (208354, Qiagen) with the LightCycler 480 Real-Time PCR System (Roche, Basel, Switzerland). The primer and probe sequences are listed in table S1.

### Inhibitor treatment on cell lines

The inhibitors including camostat, prinomastat, incyclinide, 20(R)-ginsenoside-Rh2, and E64D were obtained from Medchemexpress (Monmouth Junction, NJ, USA). Cells were treated with individual or combined inhibitors at the indicated concentrations for 2 hours at 37°C before virus infection. To test the antiviral effects of inhibitors against WT SARS-CoV-2, Calu3 and Caco2 cells were treated with individual or combined protease inhibitors at concentrations of 1, 12.5, 25, 50, and 100 μM for 2 hours before infection. Then, the treated cells were infected with WT SARS-CoV-2 at 0.5 multiplicity of infection (MOI). At 24 hpi, cell lysates and supernatants were harvested for qRT-PCR quantification of virus replication. To further evaluate the altered usage of serine proteases and MT-MMPs during SARS-CoV-2 WT and Omicron BA.1 infection, Calu3 and Caco2 cells were treated with incyclinide or camostat at 25 and 50 μM for 2 hours before infection. After the inhibitor treatments, the cells were infected with either WT SARS-CoV-2 or Omicron BA.1 at 0.5 MOI. At 48 hpi, supernatant samples were harvested for qRT-PCR quantification of virus replication. To investigate the usage of MT-MMPs by WT SARS-CoV-2 and Omicron BA.1 in 293T-ACE2 and A549-ACE2, the cells were pretreated with incyclinide, E64D, or camostat at different concentrations for 2 hours, followed by infection of either WT SARS-CoV-2 or Omicron BA.1 at 0.05 MOI. At 24 hpi, supernatant samples were harvested for qRT-PCR quantification of virus replication for both cell lines.

### Plaque assays

Plaque assays were performed as we previously described with slight modifications (*59*). Briefly, VeroE6-TMPRSS2 cells were seeded in 12-well plates at day 0. The harvested supernatant samples were serially diluted by 10-fold and inoculated to the cells for 2 hours at 37°C. After inoculation, the cells were washed with PBS three times and covered with 2% agarose/PBS mixed with DMEM/2% FBS at 1:1 ratio. The cells were fixed with 4% paraformaldehyde after 72 hours of incubation. Fixed samples were stained with 0.5% crystal violet in 25% ethanol/distilled water for plaque visualization.

### siRNA knockdown

SMARTpool ON-TARGETplus siRNA used for gene depletion was obtained from Dharmacon (Lafayette, CO, USA) (*60*). Cells were transfected with 70 nM siRNA for three consecutive days using Lipofectamine RNAiMAX (Thermo Fisher Scientific). At 24 hours after the third siRNA transfection, the cells were challenged with SARS-CoV-2 at 0.5 MOI for 2 hours at 37°C. Following virus inoculation, the cells were washed with PBS and cultured in 1% FBS DMEM/F12 (for Calu3) or 1% FBS DMEM (for Caco2) for 24 hours. At 24 hpi, the cell lysate and supernatant samples were harvested for qRT-PCR quantification of virus replication and knockdown efficiency. Infectious virus titers in the supernatant samples were determined with plaque assays.

### Cell-cell fusion assay

293T cells were transfected with SARS-CoV-2 spike as effector cells. Another population of 293T cells was cotransfected with ACE2 and target proteases as target cells. Twenty-four hours after transfection, effector and target cells were digested by EDTA-trypsin (Gibco) and mixed at a 1:1 ratio. The mixed cells were cocultured at 37°C. The number of syncytium was counted under a microscope at 24 hours after coculture.

### Western blot

Cells in 12-well plates were lysed by 125 μl of radioimmunoprecipitation assay buffer (89901, Thermo Fisher Scientific) with protease inhibitor (4693159001, Roche, Basel, Switzerland) at 24 hours after transfection. Proteins were separated by SDS–polyacrylamide gel electrophoresis gel and transferred to polyvinylidene difluoride or nitrocellulose membrane. Specific primary antibodies were incubated with the blocked membranes at 4°C overnight, followed by horseradish peroxidase (HRP)–conjugated secondary antibodies (Thermo Fisher Scientific) incubation for 2 hours at room temperature. The signal was developed by the Immobilon Crescendo Western HRP Substrate (WBLUR0500, Merck Millipore, MA, USA) and detected using the Alliance Imager apparatus (Uvitec, Cambridge, UK). Mouse anti-flag antibody (F1804, Sigma-Aldrich, St. Louis, Missouri, USA) was used as the primary antibody to detect the overexpression of the flag-tagged proteases. Human ACE2 was detected with a rabbit anti-V5 antibody (CM3005, ImmunoWay, Plano, TX, USA). β-Actin was used as the loading control, which was detected by a mouse anti-human β-actin antibody (MAB8929, R&D Systems, Minneapolis, Minnesota, USA). The spike protein was detected with a rabbit anti–SARS-CoV-2 spike S2 antibody (40590-T62, Sino Biological, Beijing, China).

### Study approval

All experiments in the study complied with the relevant ethical regulations for research. The use of animals followed all relevant ethical regulations and was approved by the Committee on the Use of Live Animals in Teaching and Research of the University of Hong Kong.

### Statistical analysis and graphics

Data represented means and SDs. Statistical differences between two groups were evaluated with two-sided unpaired Student’s *t* test using GraphPad Prism 9. Statistical differences between three or more groups were evaluated with two-way or one-way analysis of variance (ANOVA) using GraphPad Prism 9. Differences were considered statistically significant when *P* < 0.05. The figures and graphs in the manuscript were prepared with GraphPad Prism 6, Adobe Illustrator, or BioRender.com.

## Notes

View/request a protocol for this paper from *Bio-protocol*.
