# Supplementary material for: Altered host protease determinants for SARS-CoV-2 Omicron
Source: Sci Adv. 2023 Jan 20;9(3):eadd3867. doi: 10.1126/sciadv.add3867 (PMC9858505; doi:10.1126/sciadv.add3867)
Supplement: Supplementary file 1 — Figs. S1 to S12 Table S1 [file sciadv.add3867_sm.pdf]

Science Advances
AAAS

Supplementary Materials for

Altered host protease determinants for SARS-CoV-2 Omicron

Jasper Fuk-Woo Chan *et al.*

Corresponding author: Hin Chu, hinchu@hku.hk

Sci. Adv. **9**, eadd3867 (2023)
DOI: 10.1126/sciadv.add3867

This PDF file includes:

Figs. S1 to S12
Table S1

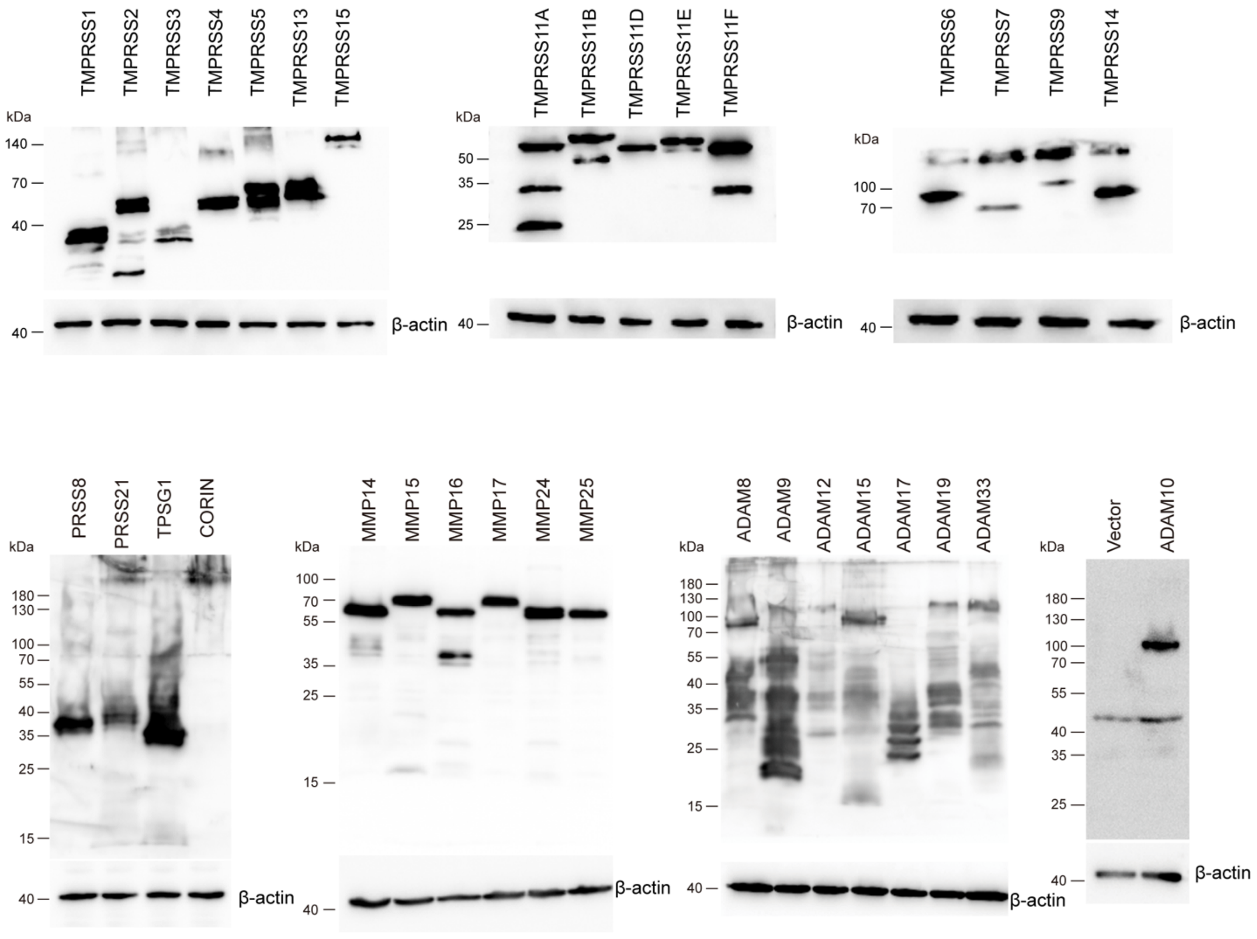

Fig S1. Protein expression of the evaluated transmembrane serine protease, MT-MMPs, and ADAMs.

293T cells were transfected with the indicated transmembrane serine protease, MT-MMPs, or ADAMs. Cell lysates were harvested at 24 hours post transfection for Western blot analysis.

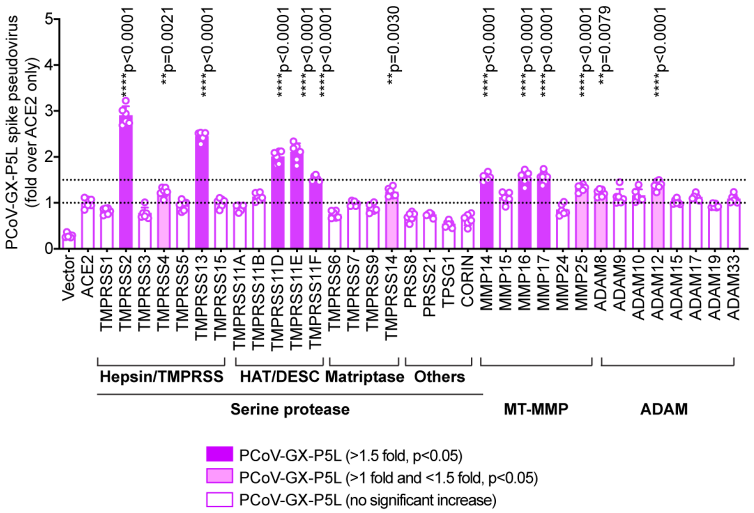

Fig S2. MT-MMPs and ADAMs facilitate pangolin coronavirus (PCoV)-GX-P5L entry.

293T cells were co-transfected with ACE2 and the indicated transmembrane serine protease, MT-MMPs, or ADAMs, and then challenged by PCoV-GX-P5L-S pseudovirus at 24 hours post transfection. Pseudovirus entry was quantified by measuring the luciferase signal of the cell lysates at 24 hours post transduction (n=6). The fold change was normalized with the ACE2 transfection group. The experiments were repeated three times independently with similar results. Data represented mean and standard deviations from the indicated number of biological repeats. Statistical significance between groups was determined with one way-ANOVA. ** represented $p < 0.01$. **** represented $p < 0.0001$.

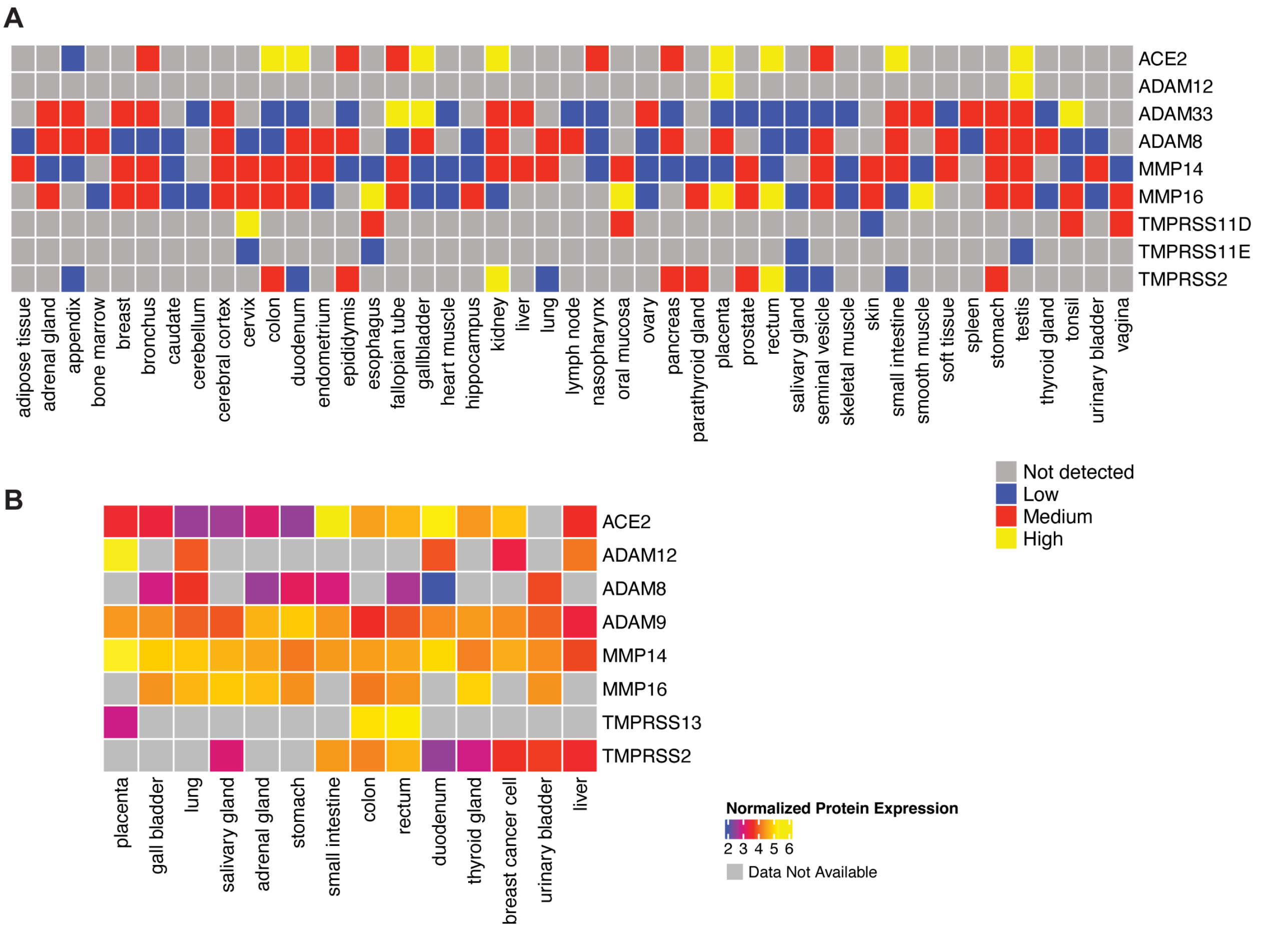

Fig S3. Proteomics analysis of ACE2 and proteases.

(A) Analysis of protein expression level of ACE2 and proteases measured by quantitative mass spectrometry. R package ggplot2 and ComplexHeatmap were used to generate the heatmap visualization. The data were downloaded from ProteomicsDB database (*50*). **(B)** Analysis of protein expression level of ACE2 and proteases measured by immunohistochemistry using tissue microarrays. R package ggplot2 and ComplexHeatmap were used to generate the heatmap visualization. Missing data are indicated by gray color. The data were downloaded from the Human Protein Atlas (http://www.proteinatlas.org) (*49*).

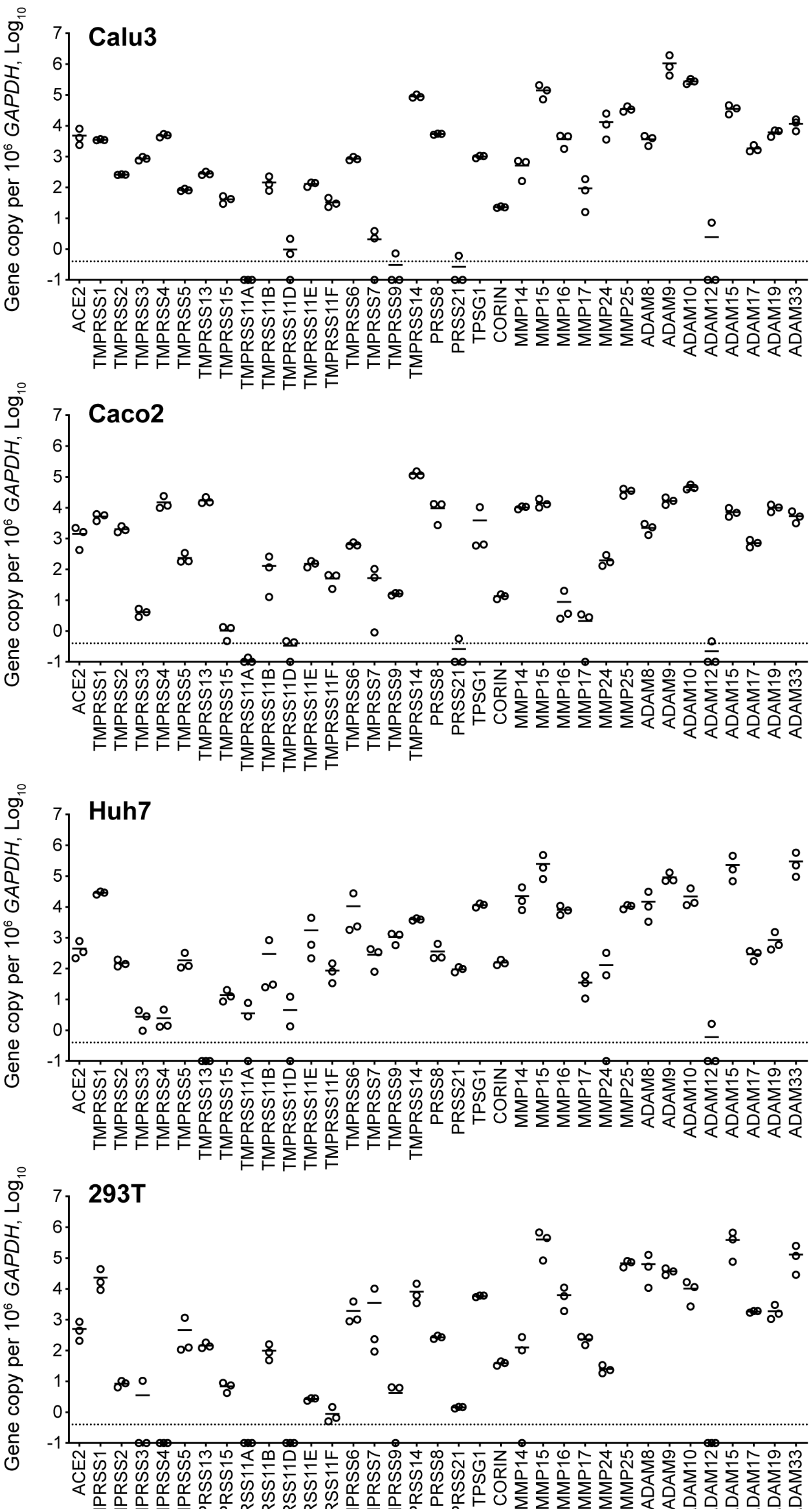
Calu3
Caco2
Huh7
293T
Gene copy per 10⁶ GAPDH, Log10
7
6
5
4
3
2
1
0
-1
ACE2
TMPRSS1
TMPRSS2
TMPRSS3
TMPRSS4
TMPRSS5
TMPRSS13
TMPRSS15
TMPRSS11A
TMPRSS11B
TMPRSS11D
TMPRSS11E
TMPRSS11F
TMPRSS6
TMPRSS7
TMPRSS9
TMPRSS14
PRSS8
PRSS21
TPSG1
CORIN
MMP14
MMP15
MMP16
MMP17
MMP24
MMP25
ADAM8
ADAM9
ADAM10
ADAM12
ADAM15
ADAM17
ADAM19
ADAM33

Fig S4. The expression of the evaluated transmembrane serine protease, MT-MMPs, and ADAMs in cell lines.

Calu3 (human lung epithelial cells), Caco2 (human intestinal epithelial cells), Huh7 (human liver cells) and 293T (human kidney epithelial cells) were lysed for RNA extraction and qRT-PCR analysis (n = 3). Gene expression was normalized to the expression of GAPDH. Data represented mean values of the indicated number of biological repeats.

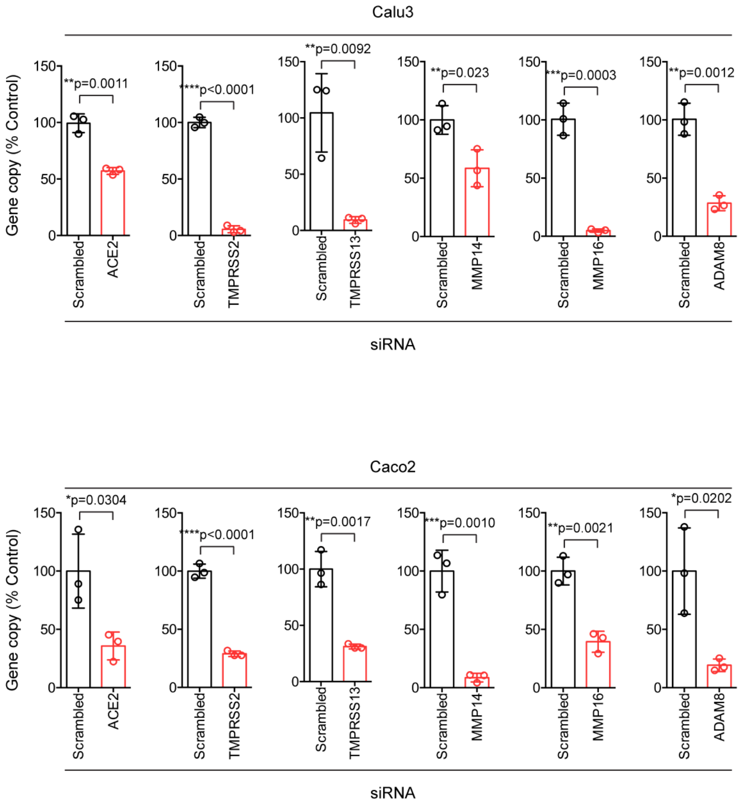

Fig S5. siRNA knockdown efficiency in Calu3 and Caco2 cells.

Calu3 and Caco2 cells were treated with ACE2, TMPRSS2, TMPRSS13, MMP14, MMP16, ADAM8, or scrambled siRNA for gene knockdown and RNA was extracted from the cell lysates for qRT-PCR analysis ($n = 3$). Data represented mean and standard deviations from the indicated number of biological repeats. Statistical significance between groups was determined with two-sided unpaired Student's t-test. * represented $p < 0.05$ and ** represented $p < 0.01$. *** represented $p < 0.001$, **** represented $p < 0.0001$.

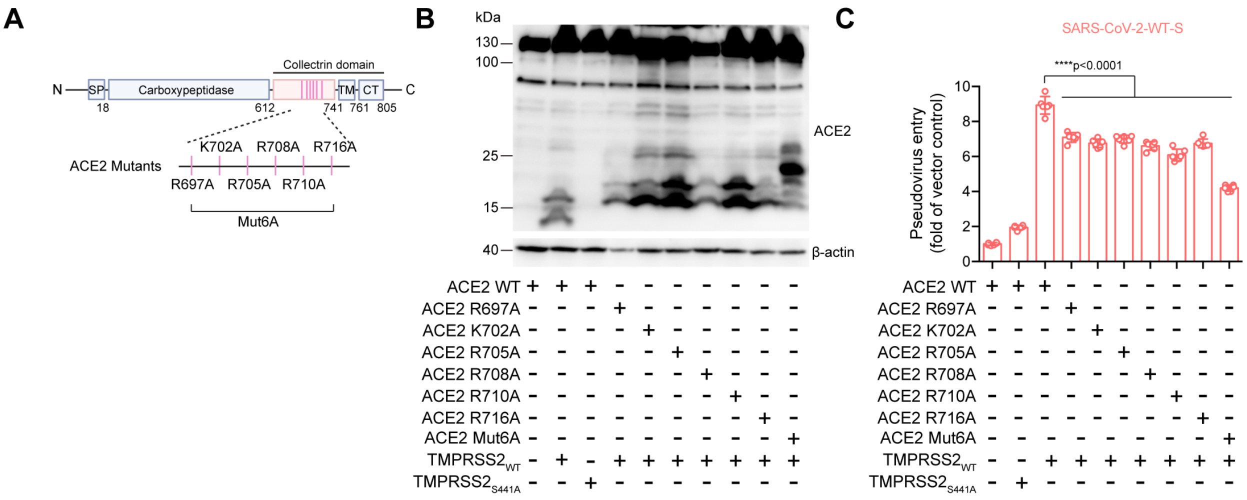

Fig S6. TMPRSS2-mediated ACE2 cleavage promotes SARS-CoV-2 entry.

(A) Schematic of the ACE2 mutants. **(B)** The cleavage of different ACE2 mutants by $TMPRSS2_{WT}$. R697A, K702A, R705A, R708A, R710A, R716A are individual cleavage site mutants located within amino acids 697 to 716 in the membrane-proximal region of hACE2, which is essential for the TMPRSS2-augmented entry of SARS-CoV-1. Mut6A contains all 6 mutations. 293T cells were co-transfected with ACE2 or ACE2 mutants and $TMPRSS2_{WT}$ or the TMPRSS2 catalytic inactive mutant ($TMPRSS2_{S441A}$). Cell lysates were harvested at 24 hours post transfection for Western blot analysis to detect ACE2 cleavage. **(C)** 293T cells were co-transfected with ACE2 or ACE2 mutants and $TMPRSS2_{WT}$ or $TMPRSS2_{S441A}$ followed by transduction with wild-type SARS-CoV-2-S (n = 6) pseudovirus. Pseudovirus entry was determined by measuring the luciferase signal of the cell lysates at 24 h post transduction. The experiments were repeated three times independently with similar results. Data represented mean and standard deviations from the indicated number of biological repeats. Statistical significance between groups was determined with one way-ANOVA. **** represented $p < 0.0001$.

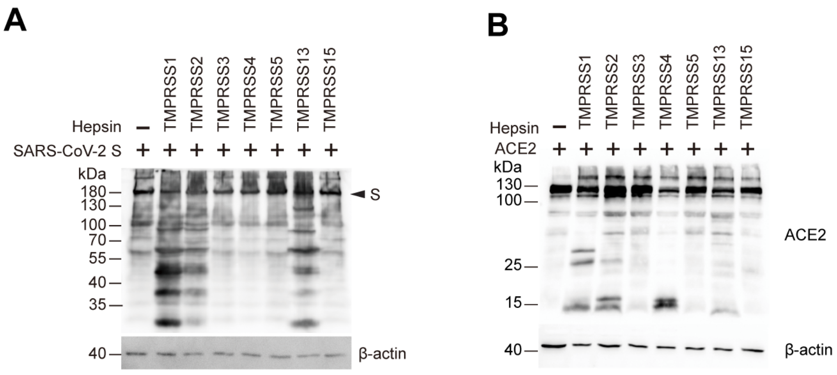

Fig S7. Cleavage of SARS-CoV-2 spike and hACE2 by the serine proteases Hepsin/TMPRSS subfamily.

293T cells were co-transfected with **(A)** SARS-CoV-2 spike and the individual proteases from the Hepsin/TMPRSS subfamily, or **(B)** ACE2 and the individual proteases from the Hepsin/TMPRSS subfamily. Cell lysates were harvested at 24 hours post transfection for Western blot analysis of SARS-CoV-2 spike and ACE2 cleavage. These experiments were repeated three times independently with similar results.

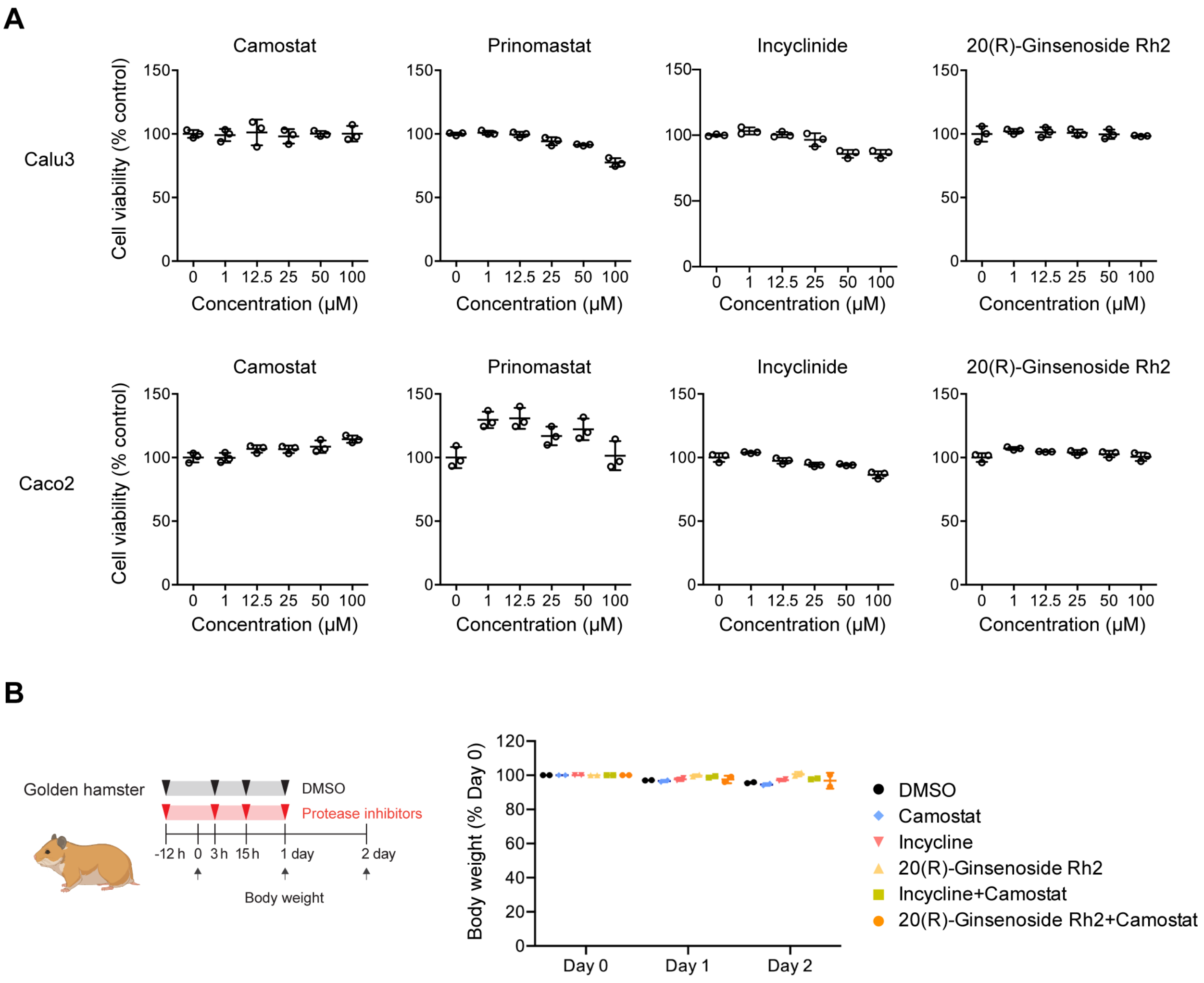

Fig S8. Assessment of pan-MMP inhibitor toxicity *in vitro* and *in vivo*.

(A) Cell viability assay. Calu3 and Caco2 cells were treated with the indicated concentrations of camostat, prinomastat, incyclinide and 20(R)-Ginsenoside Rh2 for 24 hours at 37 °C. Cell viability of the treated cells was determined by CellTiter-Glo assays (n = 3) at 24 hours post treatment. **(B)** Drug toxicity test in hamsters. Hamsters were treated with camostat, incyclinide, 20(R)-Ginsenoside Rh2, camostat+incyclinide, or camostat+20(R)-Ginsenoside Rh2 at the indicated intervals. Body weight of the animals was recorded on day 0, day 1, and day 2 post

treatment. All inhibitors did not demonstrate *in vivo* toxicity compared to the DMSO (mock)-treatment.

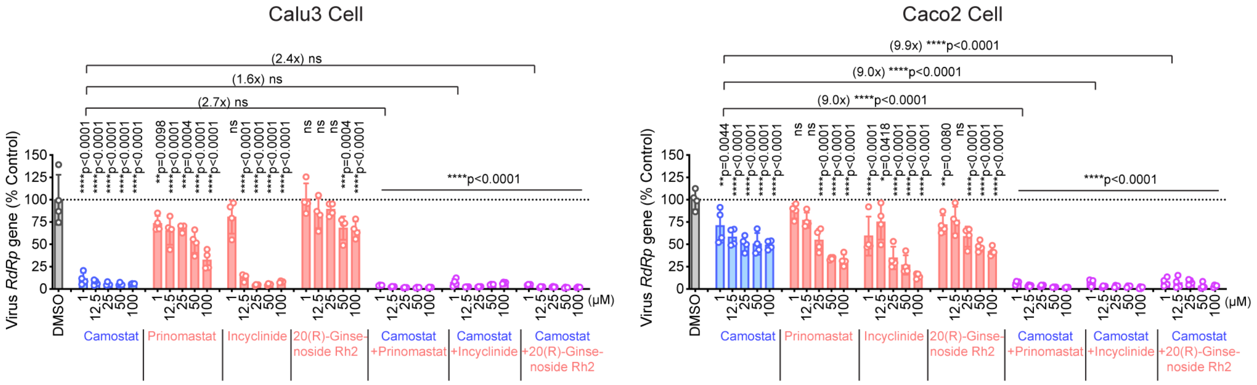

Fig S9. Pan-MMP inhibitors limit SARS-CoV-2 replication in Calu3 and Caco2 cells.

Calu3 and Caco2 cells were treated with DMSO, pan-serine protease inhibitor (camostat), pan-MMP inhibitors (prinomastat, incyclinide, 20(R)-Ginsenoside Rh2), or at the indicated combination for 2 h at 37 °C. The pre-treated cells were challenged with SARS-CoV-2 WT at 0.5 MOI for 2 h at 37 °C. Infected cells were maintained for 24 h in the presence of inhibitors. Cell lysate samples were harvested at 24hpi for qRT-PCR analysis (n = 4). Data represented mean and standard deviations from the indicated number of biological repeats. Statistical significance between groups was determined with two way-ANOVA. *represented $p < 0.05$, **represented $p < 0.01$, ***represented $p < 0.001$, **** represented $p < 0.0001$. ns = not significant.

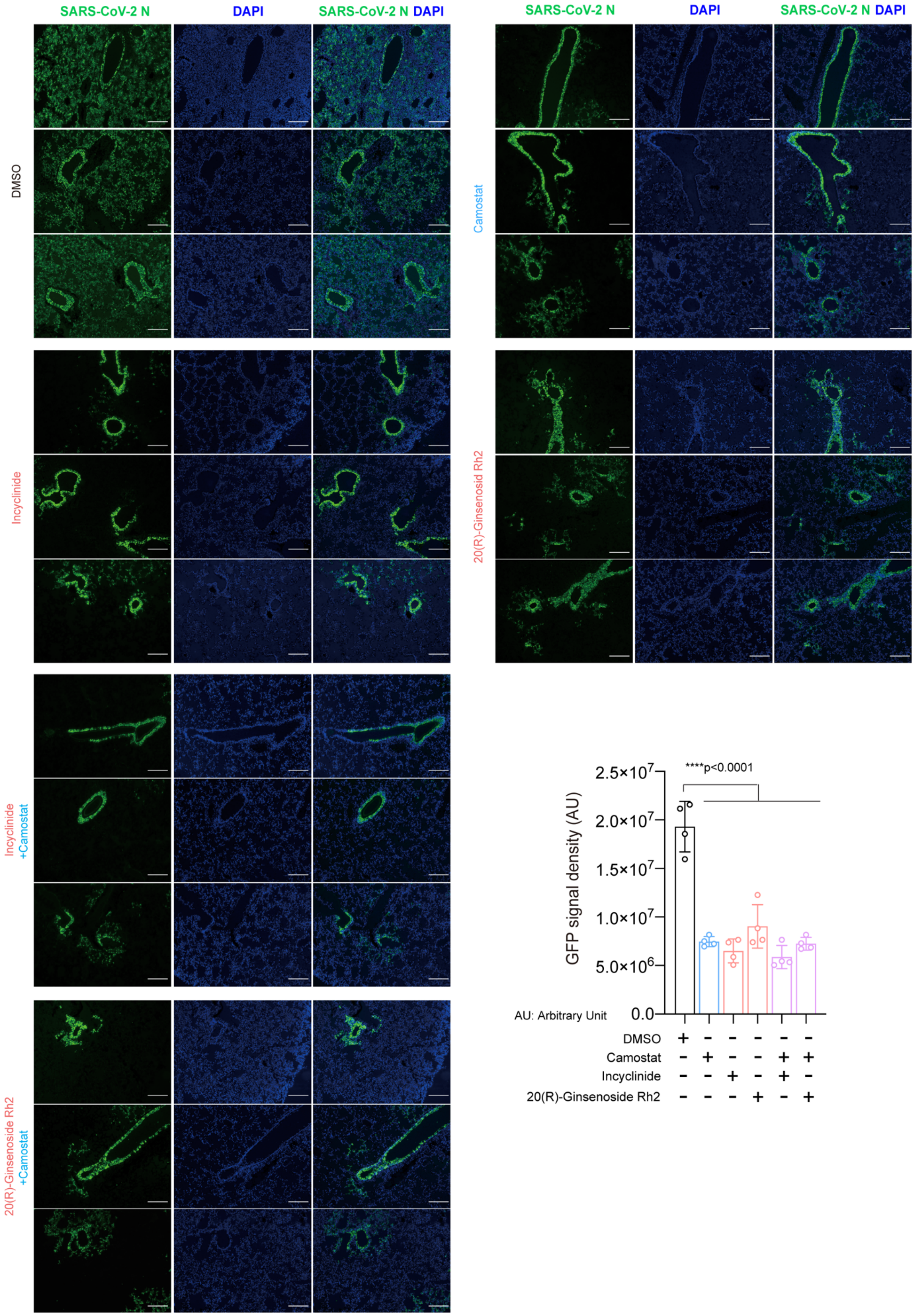
SARS-CoV-2 N
DAPI
SARS-CoV-2 N DAPI
DMSO
Camostat
Incyclinide
20(R)-Ginsenosid Rh2
Incyclinide +Camostat
20(R)-Ginsenoside Rh2 +Camostat
****p<0.0001
GFP signal density (AU)
2.5×10^7
2.0×10^7
1.5×10^7
1.0×10^7
5.0×10^6
0.0
AU: Arbitrary Unit
DMSO + - - - - -
Camostat - + - - + +
Incyclinide - - + - + -
20(R)-Ginsenoside Rh2 - - - + - +

Fig S10. Quantification of immunofluorescence antigen staining of SARS-CoV-2 WT infected hamster lungs.

Representative immunofluorescence images were demonstrated for a better overview of the inhibitory effects against SARS-CoV-2 WT by protease inhibitors. SARS-CoV-2 N protein was identified with a rabbit anti-SARS-CoV-2-N immune serum (green) and nuclei were identified with DAPI stain (blue). Bars represented 200 μm. The green fluorescent protein (GFP) signal of the representative images was quantified using ImageJ (n=4). Four fields (three demonstrated here and one in Fig 4F) were quantified for each treatment. Statistical significance between groups was determined with one way-ANOVA. **** represented $p < 0.0001$.

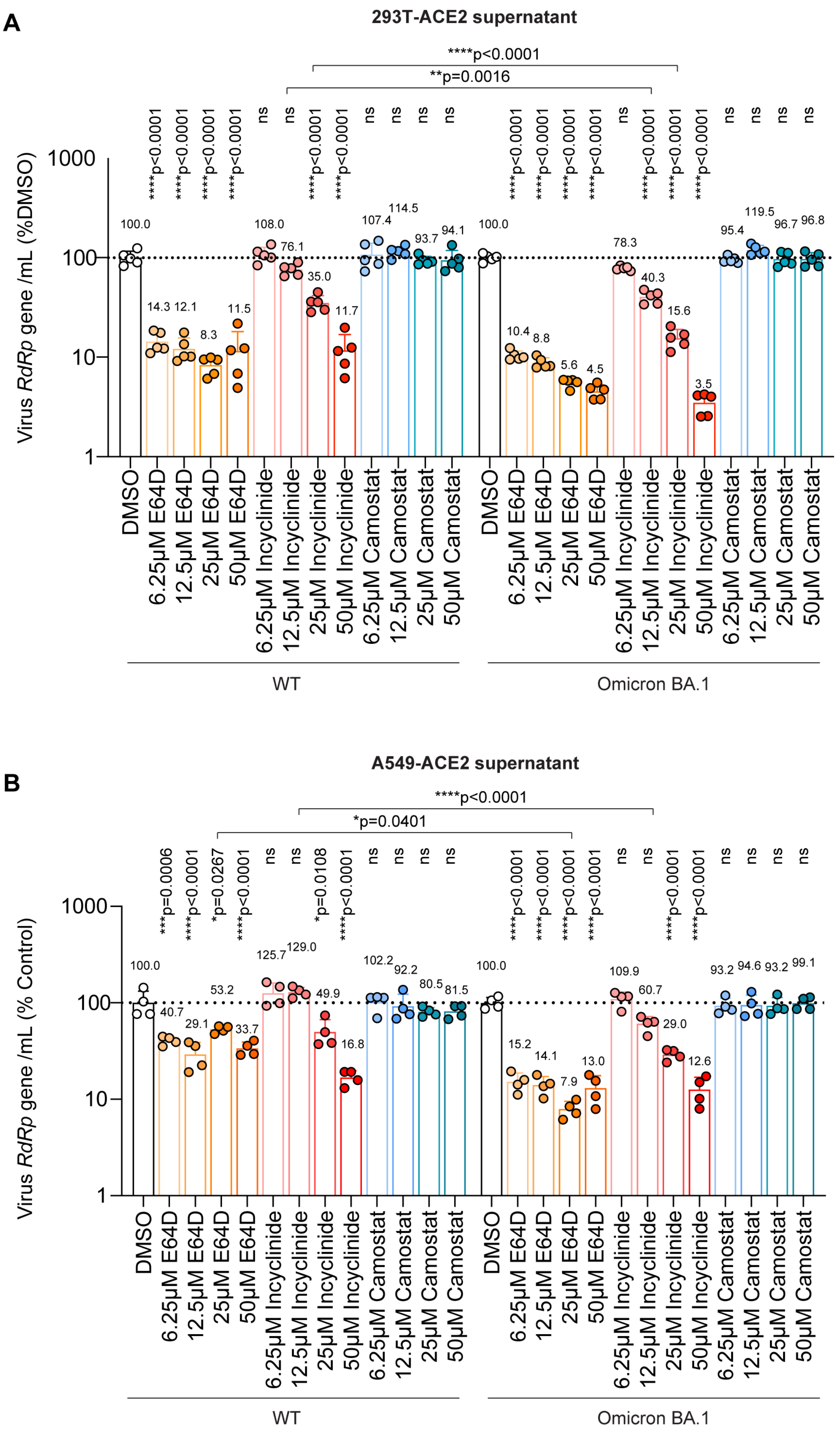
A
293T-ACE2 supernatant
****p<0.0001
**p=0.0016
Virus RdRp gene /mL (%DMSO)
1000
100
10
1
100.0
14.3
12.1
8.3
11.5
108.0
76.1
35.0
11.7
107.4
114.5
93.7
94.1
100.0
10.4
8.8
5.6
4.5
78.3
40.3
15.6
3.5
95.4
119.5
96.7
96.8
DMSO
6.25µM E64D
12.5µM E64D
25µM E64D
50µM E64D
6.25µM Incyclinide
12.5µM Incyclinide
25µM Incyclinide
50µM Incyclinide
6.25µM Camostat
12.5µM Camostat
25µM Camostat
50µM Camostat
WT
Omicron BA.1
B
A549-ACE2 supernatant
****p<0.0001
*p=0.0401
***p=0.0006
****p<0.0001
*p=0.0267
*p=0.0108
ns
Virus RdRp gene /mL (% Control)
100.0
40.7
29.1
53.2
33.7
125.7
129.0
49.9
16.8
102.2
92.2
80.5
81.5
100.0
15.2
14.1
7.9
13.0
109.9
60.7
29.0
12.6
93.2
94.6
93.2
99.1

Fig S11. Differential protease inhibitor sensitivity of SARS-CoV-2 WT and Omicron BA.1 in 293T-ACE2 and A549-ACE2 cells.

(A) 293T-ACE2 (n = 5) and **(B)** A549-ACE2 cells (n = 4) were treated with incyclinde, E64D, or camostat at the indicated concentrations for 2h at 37 °C, followed by infection of either SARS-CoV-2 WT or Omicron BA.1 at 0.05 MOI. At 24hpi, supernatant samples were harvested for qRT-PCR quantification of virus replication for both cell lines. Data represented mean and standard deviations from the indicated number of biological repeats. Statistical significance between groups was determined with two way-ANOVA. * represented $p < 0.05$, **represented $p < 0.01$, *** represented $p < 0.001$, and **** represented $p < 0.0001$. ns = not significant.

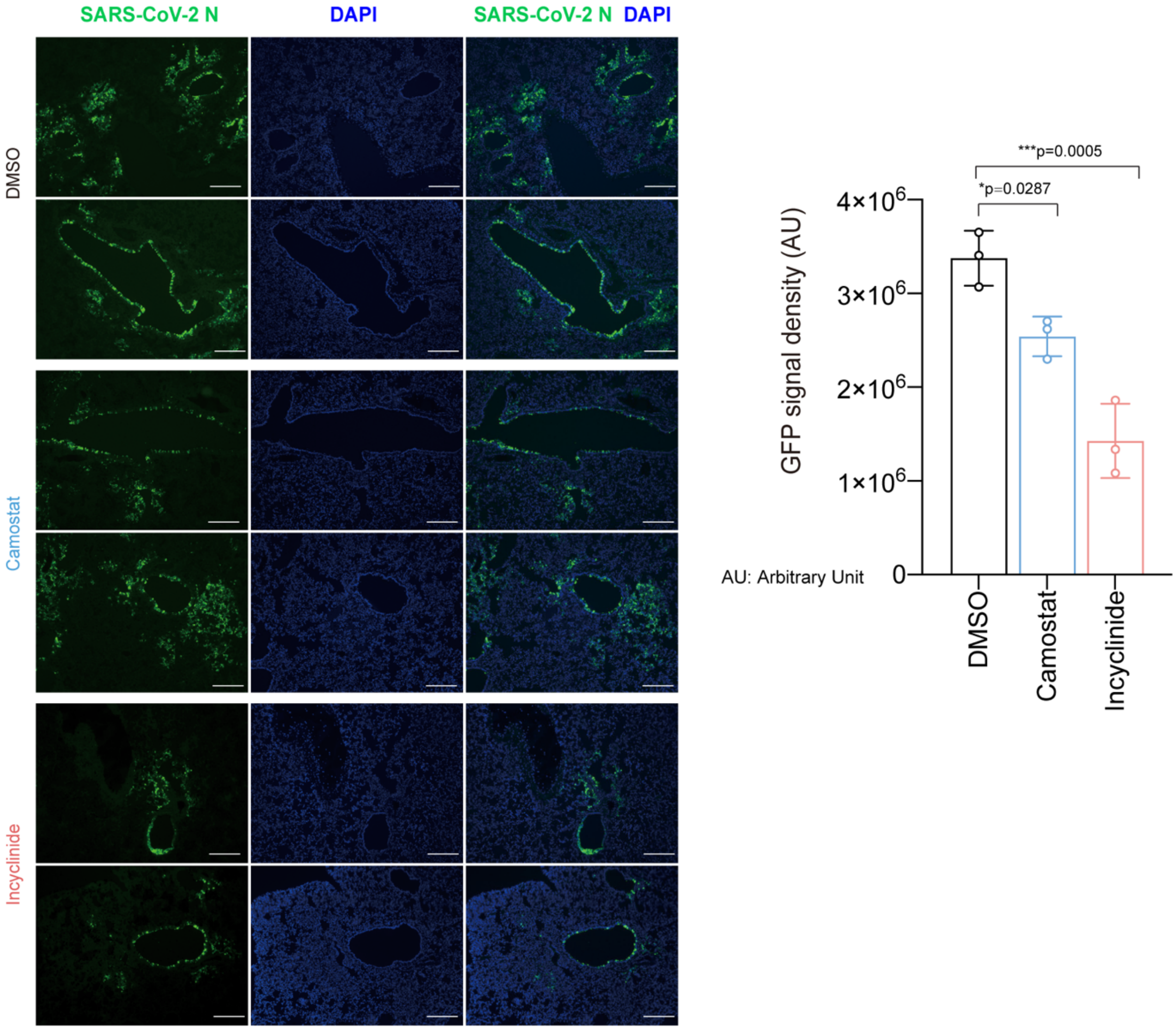

Fig S12. Quantification of immunofluorescence antigen staining of Omicron BA.1 infected hamster lungs.

Representative immunofluorescence images were demonstrated for a better overview of the inhibitory effects against Omicron BA.1 by protease inhibitors. SARS-CoV-2 N protein was identified with a rabbit anti-SARS-CoV-2-N immune serum (green) and nuclei were identified with DAPI stain (blue). Bars represented 200 µm. The green fluorescent protein (GFP) signal of the representative images was quantified using ImageJ (n=3). Three fields (two demonstrated here and one in Fig 5H) were quantified for each treatment. Statistical significance between

groups was determined with one way-ANOVA. * represented $p < 0.05$ and *** represented $p < 0.001$.

Species	Gene name	Forward Primer (5'-3')	Reverse Primer (5'-3')	Probe
Human	ACE2	CATTGGAGCAAGTGTTGGATCTT	GAGCTAATGCATGCCATTCTCA	
	PRSS8	CAGCATCACCTATGAAGGCGTC	TCCTCGGAGTAGGAGTCTAGCT	
	PRSS21	TATGCGGAGTGAGCCTGCTCAG	AGGATGGCATGGAAGTCAGCTG	
	TPSG1	GAAAGTCTCCGTGGTGGACACA	ACCGTTCACCTGGCAGACCAG	
	TMPRSS1	GTCTGCAATGGCGCTGACTTCT	TCCGAGAGATGCTGTCCTCACA	
	TMPRSS2	CTCTACGGACCAAACTTCATC	CCACTATTCCTTGGCTAGAGTA	
	TMPRSS3	GATGTGACGGAGTCTCGGATTG	TCCAGTCATCGGAGCACATGGT	
	TMPRSS4	GACGAGGAGCACTGTGTCAAGA	GAAACAGGCAGAGAACCAGTTCC	
	TMPRSS5	TGGAAGCGCAAGTGAGGGATCA	GAGGTTTACTCCCTTGTGGTGAG	
	TMPRSS6	ATCATGGCGGTCGTCTGGAAGA	CTGTTGTCCAGCGTCAGGTTCA	
	TMPRSS7	TACGACTCCCTTTTGCCCATCC	GGATTCCTGAGAGCCTCCGTAT	
	TMPRSS9	CCAGCAAGAAGTGCCTGATCTC	CACACCATCCTGTCAGTGAGTG	
	TMPRSS11A	CTCTTCCAGAGTCACCTTTTCGG	TCACTCTGGCTTCTCGGAGATC	
	TMPRSS11B	GCGGCAATCTTGGGAGTAACCA	CTGGCTTGTGAAGCTGCGTTTTC	
	TMPRSS11D	GGAGCCATCTTGTCTGGAATGC	AACCAAAGCCGCCGTGAGTCTT	
	TMPRSS11E	TTGTCCTGGCAGTGTGCATTGG	GAAGCCTCTCTGCCAAACTCAG	
	TMPRSS11F	AGTCCAGAGAGTTTGCCTCCCA	TTCCACTCTGGCTTGCCGAAGT	
	TMPRSS13	GAATGCCCTTCCCAGCGGTATA	AGTGCAGACTCACTTGCCAAGG	
	TMPRSS15	GCTTGTGCTGAGAACTGGACCA	TGGTCCACCATCGGTAGAGAAG	
	TMPRSS14	AGGACTACGTGGAGATCAACGG	TCACTGGAGTCGTAGGAGAGGT	
	MMP14	CCTTGGACTGTCAGGAATGAGG	TTCTCCGTGTCCATCCACTGGT	
	MMP15	CTGGCTCTTTCGAGAAGCGAAC	TCTCCTCGTTGAAGCGCCAGTA	
	MMP16	GATTCAGCCATTTGGTGGGAGG	CCCTTTCCAGACTGTGATTGGC	
	MMP17	CACCAAGTGGAACAAGAGGAACC	GCAATGTCGCTCCAGACCTTGA	
	MMP24	CCAGTACATGGAGACGCACAAC	TCCTCTCCGATGGTGAGTGGAT	
	MMP25	TGACAAGCCCACAAGGAAACCC	GATGGCGTCAAAATTGCCCTCAC	
	ADAM8	TGCTGGAGGTGGTGAATCACGT	TCAGGAGGTTCTCCAGTGTGAC	
	ADAM9	CTTGCTGCGAAGGAAGTACCTG	CACTCACTGGTTTTTCCTCGGC	
	ADAM10	GAGGAGTGTACGTGTGCCAGTT	GACCACTGAAGTGCCTACTCCA	
	ADAM17	AACAGCGACTGCACGTTGAAGG	CTGTGCAGTAGGACACGCCTTT	
	ADAM19	CGAGAAGGTGAATGTGGCAGGA	AGCTCTGACACTGGATCTTCCC	
	ADAM33	GGAAGAATGCCTTCCAGGAGCT	ACCAAAGCCTGGCTTGTCACAG	
	ADAM12	ATGGCATCTGCCAGACTCACGA	GGAACTCTTCGAGACTTTGCCAC	
	ADAM15	GGCACATTTGCTGCCTCGATTG	TCACACCTCCTGAGAAGTCAGG	
	GAPDH	ATTCCACCCATGGCAAATTC	CGCTCCTGGAAGATGGTGAT	
SARS-CoV-2	RdRp	CGCATACAGTCTTRCAGGCT	GTGTGATGTTGAWATGACATGGTC	FAM-TTAAGATGTGGTGCTTGCATACGTAGAC-IABkFQ
SARS-CoV-1	RdRp	CGCATACAGTCTTRCAGGCT	GTGTGATGTTGAWATGACATGGTC	FAM-CTTCGTTGCGGTGCCTGTATTAGG-IABkFQ
Hamster	β-actin	ACGGCCAGGTCATCACTATTG	CAAGAAGGAAGGCTGGAAAAG	

Table S1. Primers and probes for qRT-PCR

Primer and probes sequences used in this study were listed in this table.
